# A revision and phylogenetic analysis of *Stoiba* Spaeth 1909 (Coleoptera, Chrysomelidae)

**DOI:** 10.3897/zookeys.224.2964

**Published:** 2012-09-28

**Authors:** Chulwoo Shin, Caroline S. Chaboo

**Affiliations:** 1 Division of Entomology, 1501 Crestline Drive, Suite 140, University of Kansas, Lawrence, KS, USA 66045

**Keywords:** Chrysomelidae, Cassidinae, Mesomphaliini, *Stoiba*, Flightlessness, Cuba, Jamaica

## Abstract

*Stoiba* Spaeth, 1909 is revised with a phylogenetic analysis of 38 adult morphological characters for nine *Stoiba* species and 11 outgroup species (Mesomphaliini, Ischyrosonychini, and Hemisphaerotini). Four Cuban species of *Stoiba* were not sampled. Parsimony analysis located the four most parsimonious trees. The strict consensus (CI=0.59, RI=0.78, Steps=83) resolved the monophyly of *Stoiba*. The monophyly of *Stoiba* is supported by pale yellow antennae, antennomere VII broader than its length, and rounded basal line of pronotum. An illustrated key to ten species of *Stoiba* is provided along with a distribution map of 11 species. *Stoiba rufa* Blake is synonymized with *Stoiba swartzii* (Thunberg) by a morphological comparison which includes female genitalia.

## Introduction

*Stoiba* Spaeth 1909 was erected for *Chelymorpha flavicollis* Klug, 1829. Spaeth noted that he did not have other species on hand but that *Chelymorpha swartzii* Thunberg 1808 and *Chelymorpha angusticollis* Suffrian, 1868 might also belong in this new genus. Suffrian (1868) described *Chelymorpha fimbrialis* Suffrian and *Chelymorpha lurida* Suffrian from Cuba. Later, Borowiec (1999) classified these two as *Stoiba* species. Blake (1930) described *Stoiba bruneri* Blake, *Stoiba quatuordecimmaculata* Blake, and *Stoiba indivisa* Blake from Cuba. Later, she added *Stoiba fascicollis* Blake and *Stoiba marginata* Blake also from Cuba (Blake 1934). Zayas (1939) described *Stoiba clarildae* Zayas and *Stoiba nigricans* Zayas from Cuba. By 1946, ten *Stoiba* species were known (Blackwelder 1946).

**Figure 1.**
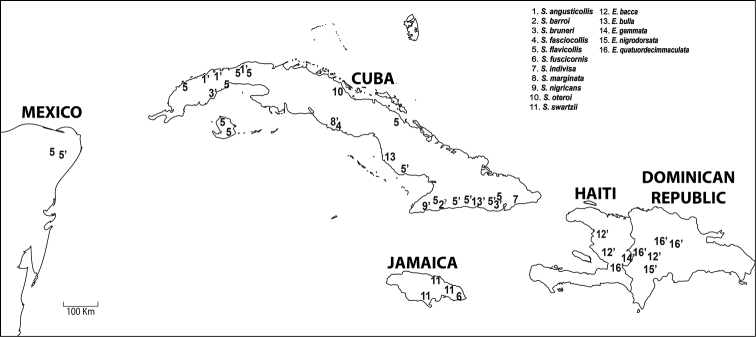
Distribution. Numbers with **’** (single quotation mark) indicate brachypterous or vestigial winged species; with ? (question mark) for species with wing status unknown.

Four more species were added in the subsequent decade — *Stoiba oteroi* Zayas and *Stoiba barroi* Zayas from Cuba (Zayas 1952), and *Stoiba fuscicornis* and *Stoiba rufa* from Jamaica (Blake 1966). Chaboo (2000) synonymized *Stoiba quatuordecimmaculata* with *Elytrogona bulla* Boheman based on a new generic definition of *Elytrogona* Chevrolat. Key diagnostic features were profile shape, elytral inflation, surface sculpture, and claw basal shape. Borowiec and Świętojańska (2012) listed 15 species in *Stoiba*. *Stoiba* and *Elytrogona* are regarded as closely related genera (Blake 1930) within the tribe Mesomphaliini because these species exhibit a range of wing development (fully developed, brachypterous, and vestigial). Their distribution is also interesting (Fig. 1). *Stoiba* occurs mainly on Cuba with the exception of four specimens of *Stoiba flavicollis* (Klug) from Yucatan, Mexico and three species on Jamaica [*Stoiba fuscicornis*, *Stoiba rufa*, *Stoiba swartzii*]. *Elytrogona* occurs only on Hispaniola with the exception of one species, *Elytrogona bulla*, from Cuba (Chaboo 2000). Chaboo (2000) found *Elytrogona* to be monophyletic based on profile shape, elytral maculation, and claw basal form (Chaboo 2000), and resolved these two genera as sister taxa in Chaboo (2007). Another phylogenetic hypothesis of Cassidinae, Hsiao and Windsor (1999), did not sample these two genera.

*Stoiba* is classified in the tribe Mesomphaliini because of this character combination: metepisternum not entirely fused with metepimeron; explanate margin of pronotum and elytra usually broad; adult head mostly hidden by pronotum; adult pronotal and elytral margin usually broad; mouthparts partly hidden by pronotum and prosternal collar; clypeus oblique and moderately long; labrum without carina; antennae filiform; pronotum without sensory setae and pores; elytra usually without sculpture, but open with large punctures; adult claws with basal tooth (Borowiec 1995). Spaeth’s (1909) generic diagnosis is based on overall body shape, but this is vague. We provide a new definition of *Stoiba* as well as notes on ten species and synonymize *Stoiba rufa* with *Stoiba swartzii*. We were unable to examine four species [*Stoiba barroi* Zayas, *Stoiba fimbrialis*, *Stoiba lurida*, and *Stoiba oteroi*] due to the restrictions in obtaining specimens from Cuban institutions. We excluded these four species from the identification key and included the English translated original descriptions in this study.

## Materials and methods

*Specimen examination*.We examined a total of 200 *Stoiba* specimens from15 museums and collections; museum acronyms (Table 1) follow Evenhuis (2012). We examined holotypes of eight species [*Stoiba bruneri* (Fig. 3), *Stoiba clarildae* (Fig. 4), *Stoiba fascicollis* (Fig. 5), *Stoiba fuscicornis* (Fig. 6), *Stoiba indivisa* (Fig. 7), *Stoiba marginata* (Fig. 8), *Stoiba nigricans* (Fig. 9), *Stoiba rufa* (Fig. 10)] from USNM, six specimens of the type series of *Stoiba flavicollis* from ZNHB (Figs 23–25), and a type specimen of *Stoiba swartzii* (Figs 38–40) from NHRS.

**Table 1.** Museums which provided specimens for the present study.

**AMNH**	American Museum of Natural History, New York, New York, U.S.A.
**BMNH**	British Museum of Natural History, London, United Kingdom.
**FMNH**	Field Museum of Natural History, Chicago, Illinois, U.S.A.
**FSCA**	Division of Plant Industry, Florida State Collection of Arthropods, Gainesville, Florida, U.S.A.
**IJSM**	Institute of Jamaica, Natural History Museum, Kingston, Jamaica.
**INHS**	Illinois Natural History Survey, Champaign, Illinois, U.S.A.
**MCZ**	Harvard University, Museum of Comparative Zoology, Cambridge, Massachusetts, U.S.A.
**MMUE**	The University of Manchester Museum, Manchester, United Kingdom.
**MZH**	Finnish Museum of Natural History, Helsinki, Finland.
**MLUH**	Martin-Luther-Universität, Wissenschaftsbereich Zoologie, Halle-Wittenberg, Germany
**NHRS**	Naturhistoriska Riksmuseet, Stockholm, Sweden
**SEMC**	University of Kansas, Snow Entomological Museum, Lawrence, Kansas, U.S.A.
**USNM**	National Museum of Natural History, Washington D.C., U.S.A.
**UWCP**	Museum of Natural History, University of Wrocław, Wrocław, Poland.
**TAMU**	Texas A & M University, College Station, Texas, U.S.A.
**ZMHB**	Museum für Naturkunde der Humboldt-Universität, Berlin, Germany.

**Figures 2–10.**
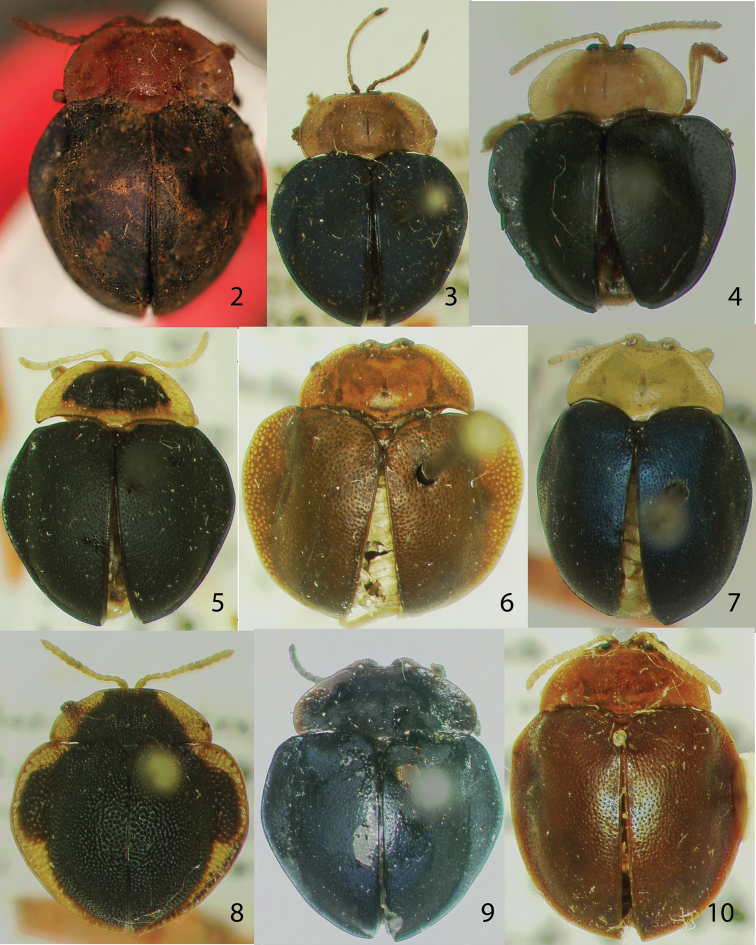
Holotypes. **2** *Stoiba barroi* **3** *Stoiba bruneri* **4** *Stoiba clarildae* **5** *Stoiba fascicollis* **6** *Stoiba fuscicornis* **7** *Stoiba indivisa* **8** *Stoiba marginata* **9** *Stoiba nigricans* **10** *Stoiba rufa* (=*Stoiba swartzii*).

Four species — *Stoiba barroi*, *Stoiba fimbrialis*, *Stoiba lurida*, *Stoiba oteroi* — were not included in the present study. *Stoiba barroi* and *Stoiba oteroi* are held by a private Cuban museum (the Zayas cassidine collection) and not available for loan. We obtained two photographs of the holotype of *Stoiba barroi* (Fig. 2) from Dr. Michael A. Ivie (Montana State University) who visited this collection.

Descriptions are based on pinned adult specimens; host plant and immature stages of *Stoiba* are unknown (Chaboo 2007; Świętojańska 2009; Borowiec and Świętojańska 2012). For the description of sexual organs, the separated abdomen parts were treated in 5–10% KOH and dissected in 95% alcohol or glycerin. Voucher dissections were preserved in glycerin. However, dissection was restricted because of limited numbers of specimens. Specimens were examined with an Olympus SZX7 microscope and an Olympus BX51 compound microscope. Measurements were made with an ocular micrometer. Photographs were taken with the Microptics® camera system. Illustrations were made with a camera lucida attached to the microscope. Terminology follows Lawrence and Britton (1991) and Chaboo (2000, 2007). We used terms “moderately convex” and “distinctly convex” to describe elytral height when the pronotum and elytra are connected continuously (Figs 15, 21, 24, 27, 30, 36, 39) or discontinuously (Figs 12, 18) in profile.

*Phylogenetic methods*.The phylogenetic analysis addressed the monophyly and species relationships of *Stoiba*. We revised the data matrix of *Elytrogona* (Chaboo 2000) and presented a new matrix for nine *Stoiba* species (Figs 11–40). Attention was paid to analyzing the morphology associated with flightlessness to determine if this arose independently in *Stoiba* and *Elytrogona*. *Stoiba clarildae* is not included because of a limited number of specimens and missing data.

*Spaethiella* sp. (Hemisphaerotini) (Fig. 81), *Asteriza flavicornis* (Olivier) (Fig. 82) and *Physonota alutacea* Boheman (Fig. 83) (Ischyrosonychini), a species from each genus *Stolas*, *Chelymorpha*, and *Phytodectoidea* (Figs 84–86), and five known *Elytrogona* species (Figs 87–91) (Mesomphaliini) were also sampled as outgroups based on Chaboo’s (2007) phylogenetic analysis. We used color of antennae as a character because it is possibly a shared character between species of *Stoiba* and *Elytrogona* (Borowiec and Świętojańska 2012). But other coloration was not included in our phylogenetic analysis because adult Cassidinae can be polymorphic in body colors, and coloration of dead specimens often does not match that of living specimens (Chaboo 2007). The data matrix of 38 morphological characters and 20 species (Tables 2–3) was created using WINCLADA (Nixon 2002), which incorporates Nona (Goloboff 1998) to analyze datasets using parsimony. All characters were equally weighted and unordered (Fitch optimization).

**Table 2.** Characters and states for the phylogenetic analysis

**Ch. No**	**Character**
0	Head, well exposed=0 (Fig. 81); mostly concealed=1 (Figs 11–40); entirely concealed=2 (Figs 82–83)
1	Antennomeres III–X, mainly pale yellow=0 (Figs 11–40); brown with black or mixed=1 (Figs 84–86)
2	Antennomere III (Figs 47–56), 2 times as long or longer than II=0; less than 2 times=1
3	Antennomere III (Figs 47–56), shorter than IV=0; as long as IV or slightly longer (1.1 times)=1; distinctly longer (over 1.2 times)=2
4	Antennomere VII (Figs 47–56), longer than width=0; broader than length or as broad as long=1
5	Mandible, teeth number five=0 (Fig. 58); 4 or 4 with vestigial teeth=1 (Fig. 59)
6	Mandible, without horizontal thickening=0 (Figs 100–101); with horizontal thickening=1 (Figs 58–59)
7	Labial palpomere I shape, sub-quadrate=0 (Fig. 102); triangular=1 (Figs 62– 63, 103)
8	Pronotum, anterior edge continuous=0 (Figs 82–91); discontinuous=1 (Fig. 81)
9	Prosternum, antero-lateral edge rounded or slightly angled=0 (Fig. 64); distinctly angled=1
10	Pronotum, base, angled=0 (Figs 84–86); transverse=1 (Figs 87–91)
11	Pronotum, widest at base=0 (Figs 82–86, 88); at middle=1 (Figs 87, 99); at front=2 (Fig. 81)
12	Pronotum, basal line, linear=0 (Figs 87–91); sinuate=1 (Figs 85–86); rounded=2 (Figs 81–83)
13	Pronotum postero-medial angle extending more than postero-lateral margin=0; (Figs 82, 84–86); not extended or slightly extended=1 (Figs 83, 87–91)
14	Prosternal process, slightly inflated=0 (Fig. 64); straight sided=1
15	Scutellum, hidden or small=0 (Figs 84, 88–91); well exposed=1 (Figs 81–83, 85–87)
16	Meso-, metanotum, separated=0 (Figs 65–66), fused=1 (Fig. 84)
17	Metasternum, medially broad and flat=0 (Fig. 67); not flat with deep groove medially=1
18	Elytral disc height/length ratio, less than 0.34=0 (Fig. 30); between 0.35-0.5=1 (Fig. 33); over 0.5=2
19	Elytra surface, smooth=0 (Figs 29, 82–87); rough=1 (Figs 11, 32, 81, 88–91)
20	Elytral puncture size, small=0 (Fig. 82–87); large=1 (Figs 81, 88–81)
21	Elytral punctuation, fine=0 (Fig. 29); rough=1 (Fig. 32); coarse (Figs 88–91)
22	Base of elytral disc, distinctly broader than discal base of pronotum=0 (Fig. 84); as broad as or slightly broader=1 (Fig. 85–86); narrower=2 (Figs 87–91)
23	Elytra, umbone indistinct=0 (Figs 87–91); distinct=1 (Figs 81–86)
24	Elytra margin, lateral edge, rounded=0 (Figs 81–87); angled=1 (Figs 88–91)
25	Elytra margin expanded toward vento-laterally=0 (81–86); horizontally=1 (87–91)
26	Elytra margin posterior half distinct from disc=0 (Fig. 23); vague=1 (Fig. 29)
27	Elytral suture, separate=0 (Fig. 35), partly fused=1 (Fig. 89), fused=2 (Fig. 88)
28	Elytral longitudinal carina and brace, parallel=0 (Fig. 96); angled=1 (Fig. 95)
29	Hind wing, fully developed=0 (Fig. 92); brachypterous=1 (Fig. 93); vestigial=2 (Fig. 94)
30	Claw basal tooth absent=0; present=1 (Figs 97–98)
31	Claw basal part, simple=0; quadrate=1 (Fig. 97); pointed=2 (Fig. 98)
32	Spermathecal duct, short=0; long=1(Figs 72–80)
33	Spermathecal receptacle, short=0, shorter than ¼ of pump (Fig. 72); long, over ¼ of pump length =1 (Fig. 73)
34	Spermathecal duct, loosely coiled=0 (Fig. 79); tightly coiled=1 (Fig. 74)
35	Male genitalia, deversment=1; without deversment=0
36	Seminal vesicle, much thicker than ejaculatory duct=0 (Fig. 99); slightly thicker=1 (Figs 68–71)
37	Aedeagal spicule, divided into two segments=0 (Fig. 99); not divided, Y or V-shaped=1 (Figs 68–71)

**Table 3.** Matrix of characters ($=polymorphism with states 0 and 1; N=not applicable)

TAXA	0	1	2	3	4	5	6	7	8	9	10	11	12	13	14	15	16	17	18	19	20	21	22	23	24	25	26	27	28	29	30	31	32	33	34	35	36	37
*Spaethiella* sp.	0	1	1	0	0	N	0	1	0	0	0	2	2	0	0	1	0	0	1	1	1	2	0	1	0	0	0	0	0	0	0	N	1	1	1	0	0	1
*Physonota alutacea*	2	1	0	2	0	0	0	0	1	0	0	0	2	1	0	1	0	0	1	0	0	0	1	1	0	0	0	0	0	0	0	N	1	1	0	1	0	0
*Asteriza flavicornis*	2	0	1	1	0	0	0	0	1	0	0	0	2	1	0	1	0	0	1	0	0	1	1	1	0	0	0	0	0	0	0	N	1	1	0	1	0	0
*Chelymorpha* sp.	1	1	0	1	0	0	1	1	1	1	0	0	1	0	0	1	0	0	0	0	0	0	1	1	0	0	0	0	1	0	1	0	1	0	0	1	1	1
*Phytodectoidea* sp.	1	1	1	1	0	0	1	1	1	1	0	0	1	0	0	1	0	0	0	0	0	0	0	1	0	0	0	0	1	0	1	1	0	0	0	1	1	1
*Stolas* sp.	1	1	0	1	0	1	1	1	1	2	1	0	0	0	0	0	1	0	0	0	0	0	0	1	0	0	0	0	1	0	0	1	0	2	0	0	?	1
*Stoiba angusticollis*	1	0	0	2	0	0	1	1	1	1	0	0	2	1	0	1	0	0	1	1	0	1	1	1	0	0	0	0	1	1	1	0	1	0	1	1	1	1
*Stoiba bruneri*	1	0	0	1	0	0	1	1	1	1	0	0	2	1	0	1	0	0	1	0	0	0	1	1	0	0	0	0	1	1	1	0	1	1	1	1	1	1
*Stoiba fascicollis*	1	0	1	1	1	0	1	1	1	1	0	0	2	1	0	1	0	0	1	0	0	0	1	1	0	0	0	0	1	0	1	0	1	0	1	1	1	1
*Stoiba flavicollis*	1	0	0	2	0	0	1	1	1	1	0	0	2	1	0	1	0	0	1	0	0	0	1	1	0	0	0	0	1	$	1	0	1	0	1	1	1	1
*Stoiba fuscicornis*	1	0	0	1	1	1	1	1	1	1	0	0	2	1	0	1	0	0	1	0	0	0	1	1	0	0	1	0	1	0	1	0	1	0	0	1	1	1
*Stoiba indivisa*	1	0	0	2	1	0	1	1	1	1	0	0	2	0	0	1	0	0	0	0	0	0	1	1	0	0	1	0	1	0	1	0	1	?	?	1	1	1
*Stoiba marginata*	1	0	0	2	0	0	1	1	1	1	0	0	2	1	0	1	0	0	1	1	0	1	1	1	0	0	0	0	1	1	1	0	1	0	0	1	1	1
*Stoiba nigricans*	1	0	0	2	1	0	1	1	1	1	0	0	2	1	0	1	0	0	1	0	0	0	1	1	0	0	0	0	1	1	1	0	1	1	1	1	1	1
*Stoiba swartzii*	1	0	0	1	1	1	1	1	1	1	0	0	2	1	0	1	0	0	0	0	0	0	1	1	0	0	0	0	1	0	1	0	1	0	0	1	1	1
*Elytrogona bacca*	1	0	0	2	1	1	1	1	1	2	1	0	0	1	1	0	1	1	2	1	1	2	2	0	1	1	0	2	0	2	1	1	0	1	0	1	1	1
*Elytrogona gemmata*	1	0	0	2	0	1	1	1	1	2	1	1	0	1	1	0	1	1	2	1	1	2	2	0	1	1	0	1	0	2	1	1	0	1	0	1	1	1
*Elytrogona nigrodorsata*	1	0	0	2	1	1	1	1	1	2	1	0	0	1	1	0	1	1	2	1	1	2	2	0	1	1	0	2	0	2	1	1	0	1	0	1	1	1
*Elytrogona quatuordecimmaculata*	1	0	0	2	1	1	1	1	1	2	1	0	0	1	1	0	1	1	2	1	1	2	2	0	1	1	0	2	0	2	1	1	0	1	0	1	1	1
*Elytrogona bulla*	1	0	0	2	0	1	1	1	1	2	1	1	0	1	1	1	1	?	2	0	0	0	2	0	0	1	0	1	0	2	1	1	1	0	1	1	1	1

## Results

### Stoiba

Spaeth, 1909: 720

http://species-id.net/wiki/Stoiba

Stoiba flavicollis [type species as originally designated], 1914: 51 [catalog]; Hincks 1952: 335 [checklist]; Jolivet 1959: 83 [locality with wing figure]; Seeno and Wilcox 1982: 174 [checklist]; Jolivet and Hawkeswood 1995: 158 [microptery]; Borowiec 1999: 130 [catalog]; Chaboo 2000: 379 [outgroup in phylogenetic analysis]; Jolivet and Verma 2002: 64 [microptery]; Chaboo 2007: 184 [phylogeny]; Borowiec and Świętojańska 2012 [online catalog].

#### Diagnosis.

*Stoiba* differs from most of mesomphaliine genera by pale antennal color (except for *Stoiba bruneri* with 11^th^ antennal segment black)and from *Elytragona* by the quadrate basal tooth of claws, rounded basal line of pronotum, separate elytral suture, moderately and distinctly convex profile, and fully-developed or brachypterous hind wing.

#### Description.

Body (Figs 11–40) generally rounded to oval with pronotum and elytra slightly to distinctly discontinuous in dorsal view, hemispherical in lateral view, widest and highest between basal 1/3 and middle of elytra.

Head (Figs 44–46) concealed by prothorax except for half of eyes and inter-ocular region in dorsal view,rounded to subquadrate (in disarticulate specimen), widest at middle, 1.25 times as broad as long; gena and eye well exposed in lateral view. Eyes large, oval, bulging, located on upper antero-lateral region of head; inter-ocular area twice as broad as eye diameter, slightly depressed or flat with antennal sockets and mid-cranial suture. Frontoclypeus (Fig. 45) broad and rounded pentagonal, rarely subquadrate with surface flat to slightly swollen (individual variation), sparsely setose; ventral angle slightly arched with frontoclypeal suture. Antenna 11-segmented (Figs 47–56), longer than lateral edge of pronotum; interantennal region as broad as antennal socket or slightly narrower; scape twice as long as broad, over 2 times longer than pedicel; pedicel as long as broad or slight longer; antennomeres III–IV slender, shiny, parallel to slightly broader apically, sparsely setose; antennomeres V–VII pale, pubescent, slightly longer or as long as broad; antennomeres VIII–X pale, as long as broad or slightly broader, pubescent setose with fine long setae on sub-apical region; antennomere IX as long as scape or slightly longer, twice longer than width, densely setose with long setae on sub-apex.

Mouth fossa (Fig. 46) rounded subquadrate with upper half broader and well-sclerotized. Labrum (Fig. 57) with basal half withdrawn into frons; anterior half shifted ventrally, sparsely punctate with long setae, broadest at shifted line with anterior edge well-sclerotized and medially emarginate. Mandible (Figs 58–59) well-sclerotized, fist-shaped with 4–5 teeth or 4 teeth with a vestigial projection ventrally; apical half shifted toward mouth fossa; basal half punctate and setose. Maxilla (Figs 60–61) long and slender; cardo long, medially narrower; stipes weakly sclerotized, irregularly triangular with short and fine setose medially; lacinia small, membranous, petal-shaped with basal region more sclerotized, densely setose; galea irregularly oval, setose with apical half more sclerotized than basal half; maxillary palpus 4-segmented with palpifer laterally connected to stipes; palpomere I shortest, triangular; II over 2 times longer than I, broader apically, slightly curved; III shorter than II and IV, broader apically with long setae on sub-apex; IV about 1.5 times as long as III, setose with apex flat and with sensilla structure. Labium (Figs 62–63) with mentum withdrawn into prosternum; ligula hemispherical with apex slightly pointed or rounded, and long setae on apical region; labial palpus 3-segmented; palpomere I triangular shorter than II and III; II as long as III, broader apically with long setae on sub-apical region; III sparsely setose with sensilla on apex.

**Figures 11–19.**
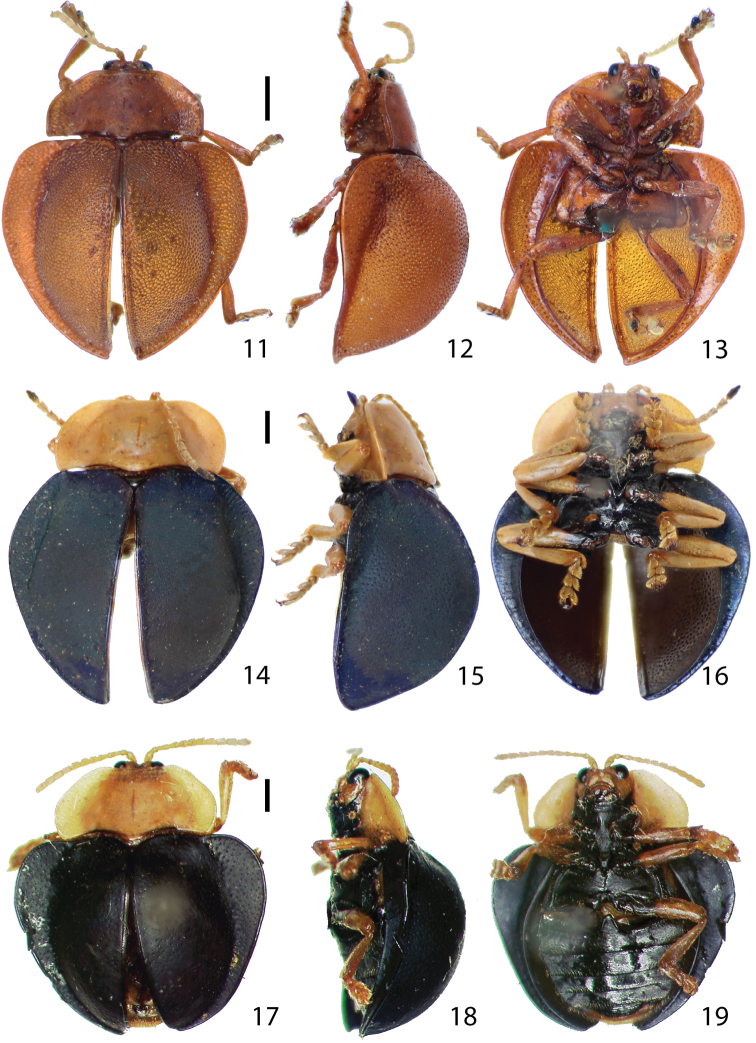
Habitus.**11–13** *Stoiba angusticollis* **11** dorsal view **12** lateral view **13** ventral view **14–16** *Stoiba bruneri* **14** dorsal view **15** lateral view **16** ventral view **17–19** *Stoiba clarildae* (Holotype) **17** dorsal view **18** lateral view **19** ventral view. (scale bar = 1.0 mm)

**Figures 20–28.**
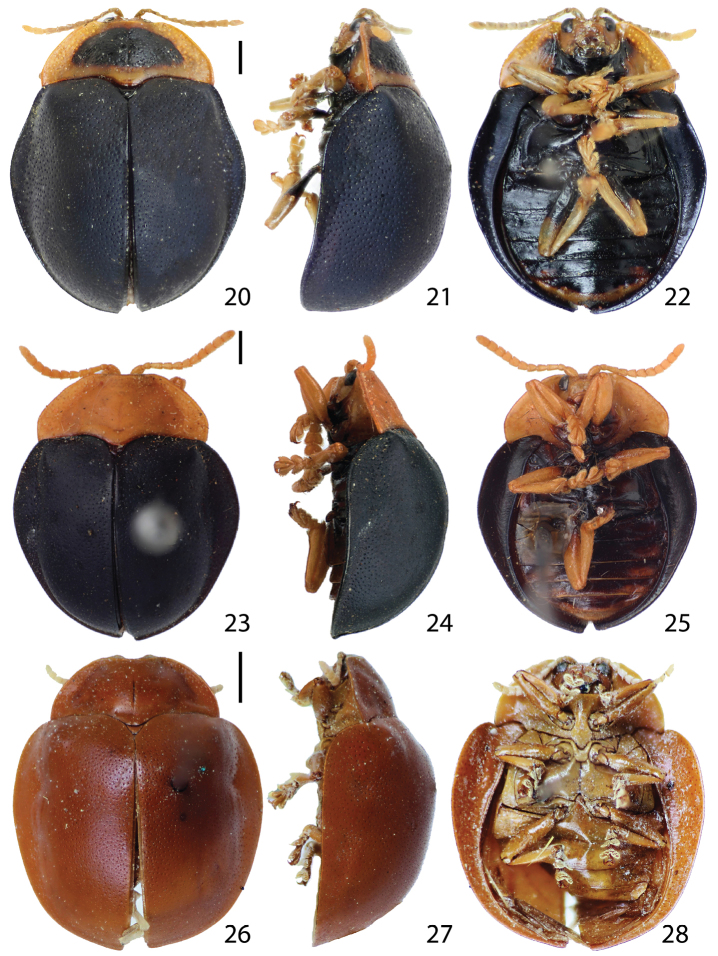
Habitus. **20–22** *Stoiba fascicollis* **20** dorsal view **21** lateral view **22** ventral view **23–25** *Stoiba flavicollis* (Syntype) **23** dorsal view **24** lateral view **25** ventral view **26–28** *Stoiba fuscicornis* **26** dorsal view **27** lateral view **28** ventral view. (scale bar = 1.0 mm)

**Figures 29–37.**
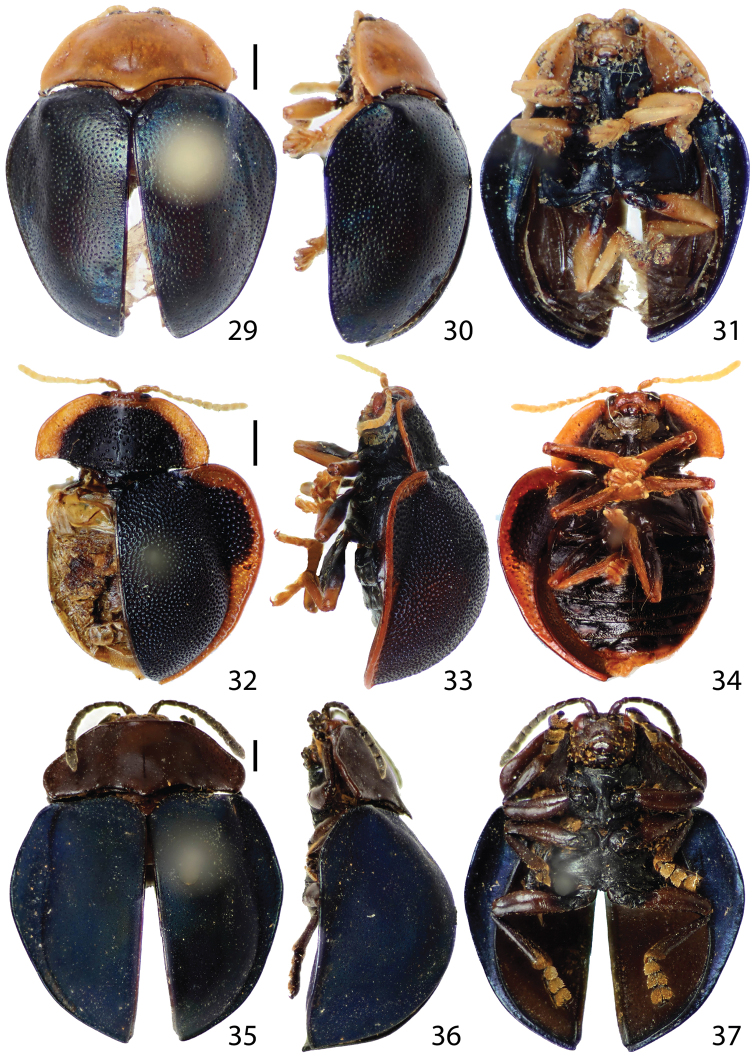
Habitus. **29–31** *Stoiba indivisa* **29** dorsal view **30** lateral view **31** ventral view **32–34** *Stoiba marginata* **32** dorsal view **33** lateral view **34** ventral view **35–37** *Stoiba nigricans* **35 **dorsal view **36** lateral view **37** ventral view (scale bar = 1.0 mm)

**Figures 38–43.**
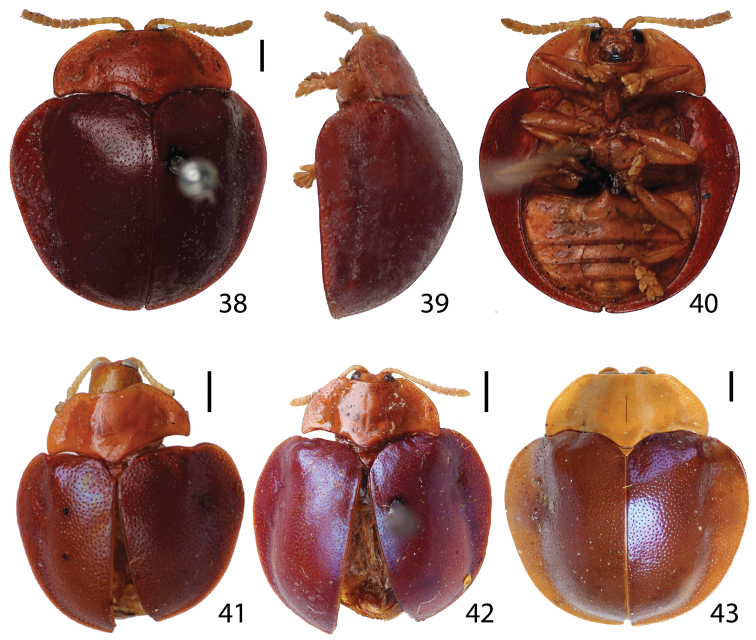
Habitus. **38–40** *Stoiba swartzii* (Holotype) **38** dorsal view **39** lateral view **40** ventral view **41** paratype of *Stoiba rufa* (=*Stoiba swartzii*) **42–43** *Stoiba swartzii*. (scale bar = 1.0 mm)

Pronotum (Figs 11, 14, 17, 20, 23, 26, 29, 32, 35, 38) hemispherical or trapezoidal in dorsal view with anterior edge linear or slightly emarginate, broadest between middle and base; base broadly rounded with postero-medial edge extended, covering anterior half of scutellum; disc with explanate margin distinct or posterior half indistinct, smooth [except for *Stoiba fascicollis* (Fig. 20) and *Stoiba marginata* (Fig. 32)], slightly convex with longitudinal cleavage medially; margin area broader basally, slightly inclined upward, weakly punctate (except for some *Stoiba flavicollis* specimens withsmooth surface); profile irregularly trapezoidal, highest at base; lateral edge rounded to slightly angled with postero-lateral edge slightly extended.

Prosternum (Fig. 64) flat or slightly convex; anterior prosternal edge linear to slightly curved, forming cervical cavity; prosternal process smooth or occasionally with shallow depression, reaching mesocoxae with arrow-shaped apex.

Mesonotum (Fig. 65) transverse with basal edge line well-sclerotized, weakly fused to metanotum; scutellum well-sclerotized, triangular, convex with anterior half withdrawn into pronotal base.

Mesosternum (Fig. 67) deeply notched; mesepisternal ridge well defined; mesosternal process extended to posterior end of mesocoxal cavity, fused to metasternum.

Metanotum (Fig. 66) weakly sclerotized; scutellar groove and scutoscutellar suture distinct. Metasternum (Fig. 67) flat or slightly convex medially with distinct longitudinal line, transverse posteriorly, laterally declined; intercoxal notch distinct, as long as or slightly shorter than hind trochanter.

Elytra (Figs 11, 14, 17, 20, 23, 26, 29, 32, 35, 38) oval to round with base transverse, explanate laterally, moderately to distinctly convex; outline slightly to moderately discontinuous between anterior 1/3 and middle in dorsal view; surface fine scaled-like, shining to murky, finely to coarsely or roughly punctate; punctures evenly sized; brace (Fig. 95) distinct with posterior end weakly connected to longitudinal carina forming angle; color various; margin area broadest between anterior 1/3 and middle, gradually narrower posteriorly. Hind wing (Figs 92–93) fully developed or brachypterous.

Legs (Figs 13, 16, 19, 22, 25, 28, 31, 34, 37, 40) slender, long, shiny, brown to black, extending beyond elytral margin; trochanters short, triangular; femora moderately broad, broadest at middle, much narrower toward base rather than distal end; tibiae as long as femur or slightly shorter, slightly broader apically with apical end notched, broadest at notched region; distal 1/3 (lateral areas of notched region) coarsely setose; with tarsomeres I, II, III, IV dorsally convex with long setae; ventral surface densely setose, pale; tarsomere I small, rounded triangular; II with apex slightly bilobed, 2 times as long as I; III deeply bilobed, *ca*. 3 times as long as I; IV 3 times as long as tarsomere I, slightly broader apically, covering base of claws; claws evenly curved, tapered with quadrate basal tooth.

Abdomen (Figs 13, 16, 19, 22, 25, 28, 31, 34, 37, 40) fully covered by elytra, broad, rounded laterally, slightly convex medially; each ventrite well-sclerotized, same size in length (without hind coxal process on ventrite I), sparsely setose with posterior and lateral areas more setose; ventrite V much narrower than I–IV with longer setae.

Aedeagus (Figs 68–71) curved in lateral view (only *Stoiba flavicollis* and *Stoiba swartzii* dissected), laid laterally (male genitalia deversement) with aedeagal base piece oval, membranous structure basally, slightly broader apically with apex pointed; tegmen sclerotized, Y-shaped; spicule V-shaped with anterior end slightly extended; ejaculatory duct longer than base piece; seminal vesicle slightly longer than base piece with sclerotized bead between ejaculatory duct and seminal vesicle.

Spermatheca (Figs 72–80) irregular falcate or irregular J-shaped to C-shaped, short to elongated; receptacle distinct from pump with two openings; spermathecal duct various in shape and length, loosely coiled to moderately coiled or entwined.

#### Remarks.

We found some morphological variation especially in body shape, pronotum, and elytra. From the dissected or sex-determined specimens we considered those variations in males and females, but could not find any sexual dimorphism.

#### Key to species

**Table:** 

1	Elytral disc mainly black to dark blue (Figs 14, 17, 20, 23, 29, 32, 35)	2
–	Elytral disc brown to reddish brown with or without blue metallic tint (Figs 11, 26, 38)	8
2(1)	Pronotum bicolored (Figs 20, 32); pronotal disc black with margin area brown to reddish brown; prosternal process black with black coloration extending to hypomeron; meso-, metafemur (Figs 22, 34) with more than proximal 1/3 black	3
–	Pronotum unicolored (brown or pitchy brown; Figs 14, 17, 23, 29, 35); prosternal process black with hypomeron brown to dark brown; each femur brown with less than proximal 1/3 black (Fig. 19, 19, 25, 31, 37)	4
3(2)	Elytra and pronotum (Fig. 20) finely punctate with surface scale-like; elytra unicolored (black or dark blue); prosternum (Fig. 22) black with black coloration extending to antero-lateral area of hypomeron; elytral base as wide as pronotal base (Fig. 20); hind wing fully developed; meso-, metafemur up to half black (Fig. 21)	*Stoiba fascicollis* Blake
–	Elytra and pronotum (Fig. 32) coarsely and roughly punctate; prosternum (Fig. 34) black with black coloration extending to middle of hypomeron; elytra (Fig. 32) bicolored with disc black; black coloration extending to middle of elytra margin area, surrounded by brown margin area; elytral base (Fig. 32) distinctly broader than pronotal base; hind wing brachypterous (Fig. 32); femora (Fig. 34) with more than proximal half black	*Stoiba marginata* Blake
4(2)	Pronotum (Fig. 35) pitchy brown or black; antennae, thoracic sterna, coxae, tarsi black with head, femora, tibiae blackish brown (Fig. 37); hind wing brachypterous	*Stoiba nigricans* Zayas
–	Pronotum (Figs 14, 17, 23, 29) brown to reddish brown; thoracic sterna, coxae, trochanters, distal region of femora brown to reddish brown; proximal end of femora black (Figs 16, 19, 25, 31); hind wing fully developed or brachypterous	5
5(4)	Pronotum hemispherical without angle at posterior 1/3 of lateral edge; elytra (Fig. 29) dark blue to purple; margin in posterior half narrow, indistinct (Fig. 29); hind wing fully developed	*Stoiba indivisa* Blake
–	Pronotum trapezoidal, rarely hemispherical; elytra (Figs 14, 17, 23) black to bluish black; margin in posterior half wide, distinct; hind wing brachypterous or fully developed	6
6(5)	Antennomere XI pale (Figs 4, 17, 23)	7
–	Antennomere XI black (Figs 3, 14, 48)	*Stoiba bruneri* Blake
7(6)	Body distinctly convex (Fig. 18); elytral base much broader than pronotal base (Fig. 17); elytra broadest near basal 1/3, black without metallic luster	*Stoiba clarildae* Zayas
–	Body moderately convex (Fig. 24); elytral base as broad as pronotal base or slightly broader than pronotal base (Fig. 23); elytra broadest near middle, brownish black to black with weak metallic luster	*Stoiba flavicollis* (Klug)
8(1)	Body (Fig. 12) distinctly convex; mandible (Fig. 58) with 5 teeth; pronotum and elytra (Figs 11–12) opaque; elytra (Fig. 11) coarsely and roughly punctate; collected from Cuba	*Stoiba angusticollis* (Suffrian)
–	Body (Figs 27, 39) moderately convex; mandible (Fig. 59) with 4 teeth or 4 teeth with additional small vestigial tooth ventrally; elytra (Figs 26, 38) finely punctate; collected from Jamaica	9
9(8)	Body (Fig. 26) unicolored (leathery brown to reddish brown); posterior half of explanate margin not well defined	*Stoiba fuscicornis* Blake
–	Pronotum and elytra (Fig. 38) shiny; elytra opalescent, always darker (or more reddish) than pronotum; elytral disc moderately defined	*Stoiba swartzii* (Thunberg)

### Stoiba angusticollis

(Suffrian, 1868)

http://species-id.net/wiki/Stoiba_angusticollis

Figures 11–13

Chelymorpha angusticollis Suffrian 1868: 239; Gemminger and Harold 1876: 3638 [catalog]; Leng and Mutchler 1914: 458 [list of the West Indies Coleoptera].
Stoiba angusticollis : Spaeth 1909: 720 [catalog], 1914: 51 [catalog]; Blackwelder 1946: 743 [checklist]; Wilcox 1975: 151 [checklist]; Borowiec 1996: 229 [faunistic record], 1999: 130 [catalog]; Takizawa 2003: 105 [checklist]; Borowiec and Świętojańska 2012 [online catalog].

#### Type material.

Unknown.

#### Type locality.

“Cuba”

#### Specimens examined.

Cuba: Prov. La Havana: ex FC Bowditch collection (MCZ: 2); ex F Monrós collection (USNM: 1); no further data (AMNH: 2; MCZ: 1; MMUE: 1; USNM: 1); Pinar del Rio: Pan de Guajaibon, May 17 1953, MJ Jaume (INHS: 1); Soroa July 6–7 1974, Z&M Mészáros (UWCP: 1).

#### Diagnosis. 

*Stoiba angusticollis* is one of three species with brown tone elytra. This species is easily distinguished from the other two *Stoiba* species (*Stoiba fuscicornis* and *Stoiba swartzii*) with brown elytra by body shape, dorsal surface coarsely and roughly punctate, opaque coloration, mouth parts, and collecting locality. Body distinctly (Fig. 12) convex; dorsal surface coarsely and densely punctate (Fig. 11), opaque brown; mandible (Fig. 58) with 5 teeth; maxillary palpus (Fig. 60) and labial palpus (Fig. 62) compact; collection locality in Cuba.

#### Description.

Adult (n=10) length 6.1–8.0 mm, width 6.0–7.8 mm. Body (Figs 11–13) rounded with elytral base broader than pronotal base; profile (Fig. 12), 0.5 times as convex as long, often discontinuous between pronotal and elytral bases, highest at anterior 1/3 of elytra; color brown to reddish brown, opaque; surface texture scale-like, roughly and coarsely punctate. Antenna (Figs 11, 47) as long as or slightly longer than pronotal lateral edge; antennomeres I–II reddish brown, shiny, darker apically; III long, broader apically, 2 times longer than II, 1.2 times longer than IV; V–XI pubescent with long setae on apex. Mandible with 5 teeth; maxillary palpus and labial palpus compact. Pronotum (Fig. 11) hemispherical with anterior margin slightly emarginate; disc slightly convex with longitudinal cleavage on surface medially. Elytra distinctly convex, finely and more coarsely punctate than pronotum. Spermatheca (Fig. 72) falcate with two openings; receptacle 0.2 times as long as pump; spermathecal duct moderately coiled.

#### Distribution.

Cuba: Prov. La Havana; Pinar del Rio.

#### Remarks.

Our description was made by observing the examined specimens and comparing to the original description (Suffrian 1868). The original description is sufficient to recognize the species with features such as convex body, coloration, and collecting location. The description corresponds to our observation.

### Stoiba barroi

Zayas, 1952

http://species-id.net/wiki/Stoiba_barroi

Figure 2

Stoiba barroi Zayas 1952: 72 [original description including figure]; Wilcox 1975: 151 [checklist]; Borowiec 1999: 130 [catalog]; Takizawa 2003: 105 [checklist]; Borowiec and Świętojańska 2012 [online catalog].

#### Type material.

Holotype (Fig. 2).

**Type locality.** Cuba: Prov. Granma or Santiago de Cuba: Sierra Maestra, between Loma Pico Palma Mocha and Pico Biscupe de Joaquín, 3500 ft (see “Remarks” portion below).

#### Diagnosis

**(from original description by Zayas 1952).** *Stoiba barroi* is the largest among the Cuban species, similar to *Stoiba flavicollis* but separated by coloration of antennae, head, and legs. *Stoiba barroi* is also similar to *Stoiba bruneri* but distinguished by the color of the elytra, which is clearly blue, and the colors of head and legs, which are also conspicuous. Compared to *Stoiba clarildae*, *Stoiba barroi* is more elongated and differs in colors of head, legs, and antennae.

#### Description

**(from original description by Zayas 1952).** Adult length 10.0 mm, width 8.0 mm. Body robust, strongly convex with antennomere XI and elytra matte black; legs dark ferruginous; lateral margin of pronotum dark yellow, depressed and convex at edge. Head concealed in dorsal view. Pronotum broadly expanded, lateral margin very upturned with well-marked transverse furrow between pronotal disc and scutellum; surface finely wrinkled with scattered punctures, faintly printed; wrinkles almost imperceptible on discal area, conspicuous at the edges. Elytra strongly convex with expanded margins and elytral suture significantly lifted; surface slightly rough as on pronotum and more densely and roughly punctate.

#### Distribution.

Cuba: Prov. Granma or Santiago de Cuba.

#### Remarks.

According to the photograph of the holotype of *Stoiba barroi* (Fig. 2), it is easily distinguished from *Stoiba bruneri* by brown antennomere XI (black in *Stoiba bruneri*). The locality information in the original description (Zayas 1952) is not precise enough to define the province. We indicated two provinces (Granma or Santiago de Cuba) based on the collecting locality data.

### Stoiba bruneri

Blake, 1930

http://species-id.net/wiki/Stoiba_bruneri

Figures 3 14–16

Stoiba bruneri Blake 1930: 219 [original description including figure]; Blackwelder 1946: 743 [checklist]; Wilcox 1975: 151 [checklist]; Borowiec 1999: 130 [catalog]; Chaboo 2000: 379 [outgroup in phylogenetic analysis]; Takizawa 2003: 105 [checklist]; Borowiec and Świętojańska 2012 [online catalog].

#### Type material.

Holotype (Fig. 3) and four paratypes in USNM (Type No. 43117).

#### Type locality.

Cuba: Prov. Guantánamo: Sierra Maestra Palma Mocha 1386 m.

#### Specimens examined.

Cuba: Prov. Guantánamo: Sierra Maestra July 10–20 1922, CH Ballou, SC Bruner, Palma Mocha 1386 m. (USNM: holotype, male; paratype, type No. 43117); Sierra Maestra 900–1200 m, July 10–20 1922, CH Ballou, SC Bruner, EEA. de Cuba, No. 9355, (USNM: paratype, type No. 43117); Sierra Maestra July 10–20 1922, CH Ballou, SC Bruner, 4000–5000 ft. EEA. de Cuba, No. 9355, (USNM: 2 paratypes, type No. 43117); Sierra Maestra, Pico Joaquin, 5300 ft. May 18 1948, J Ferras (USNM: 1 female, MCZ: 1).

#### Diagnosis.

*Stoiba bruneri* (Figs 14–16) is similar to *Stoiba clarildae*, *Stoiba flavicollis* and *Stoiba indivisa* but it is mainly distinguished by the black antennomere XI and more rounded lateral edge of the pronotum. It is also distinguished from *Stoiba clarildae* by its shiny black scutellum and brachypterous hind wing and from *Stoiba indivisa* by the distinct posterior half of the elytral margin and brachypterous hind wing.

#### Description.

Adult (n=7) length 7.0–8.5 mm, width 7.0–8.5 mm. Body (Fig. 14–16) rounded, as long as wide, discontinuous between pronotum and elytra, broadest at anterior 1/3 of elytra in dorsal view; profile moderately convex, highest at middle. Antennae pale with antennomere XI black; antennomere III slightly broader apically, almost as long as IV (Fig. 48). Mandible (Fig. 58) with 5 teeth; maxillary palpus (Fig. 60) and labial palpus (Fig. 62) compact. Pronotum (Fig. 14) brown to dark brown, hemispherical with anterior margin slightly emarginate, laterally rounded, slightly convex with surface smooth; margin area distinct in anterior half, vague in posterior half, shallowly and sparsely punctate. Elytra (Fig. 15) moderately convex, blue to dark blue, finely and moderately punctate, broadest at anterior 1/3; margin area distinctly narrower posteriorly, not extending to terminal end. Spermatheca (Fig. 73) falcate with two openings, receptacle 0.5 times as long as pump.

#### Distribution.

Cuba: Prov. Guantánamo.

### Stoiba clarildae

Zayas, 1939

http://species-id.net/wiki/Stoiba_clarildae

Figures 4 17–19

Stoiba clarildae Zayas 1939: 253 [original description including figure]; Blackwelder 1946: 743 [checklist]; Wilcox 1975: 151 [checklist]; Borowiec 1999: 130 [catalog]; Takizawa 2003: 105 [checklist]; Borowiec and Świętojańska 2012 [online catalog].

#### Type material.

Holotype (Figs 4, 17–19) in USNM (Type No. 43117).

#### Type locality.

Cuba: Prov. Guantánamo: Baracoa, El Yunque.

#### Specimens examined.

Cuba: Prov. Guantánamo: Baracoa, El Yunque, July 1935 (USNM: holotype, type No. 53529).

#### Diagnosis.

*Stoiba clarildae* is similar to *Stoiba bruneri* and *Stoiba flavicollis*, but it differs from *Stoiba bruneri* by pale, unicolored antennae, less explanate pronotal margin, shiny, dark brown scutellum, and fully developed hind wing. It differs from *Stoiba flavicollis* by black elytra without metallic luster, pronotum shape, elytral base much broader than pronotal base, and elytra broadest near basal 1/3.

#### Description.

Adult (n=1) length 7.7 mm, width 7.6 mm. Body (Figs 17–19) rounded, as long as wide greatly discontinuous between pronotum and elytra in dorsal view; profile distinctly convex, broadest and highest at anterior 1/3 of elytra. Head and pronotum brown; antennae pale brown; scutellum shiny, dark brown; prosternal process black with hypomeron brown; thoracic sterna, coxae, trochanters, proximal 1/3 of femora black; elytra black. Mandible (Fig. 58) with 5 teeth; maxillary palpus (Fig. 60) and labial palpus (Fig. 62) compact. Pronotum (Fig. 17) hemispherical with anterior margin slightly emarginate; lateral edge rounded, slightly angled at middle; pronotal disc slightly convex with surface scale-like, sparsely and finely punctate; explanate margin moderately distinct, shallowly and sparsely punctate. Elytra (Fig. 18) distinctly convex; surface scale-like, finely and moderately punctate, broadest at anterior 1/3; margin area narrower posteriorly, extending to terminal end.

#### Distribution.

Cuba: Prov. Guantánamo.

### Stoiba fascicollis

Blake, 1934

http://species-id.net/wiki/Stoiba_fascicollis

Figs 5 20–22

Stoiba fascicollis Blake 1934: 54 [original description including figure]; Blackwelder 1946: 743 [checklist]; Wilcox 1975: 151 [checklist]; Borowiec 1999: 130 [catalog]; Takizawa 2003: 105 [checklist]; Chaboo 2007: 28 [figure I misnamed]; Borowiec and Świętojańska 2012 [online catalog].

#### Type material.

Holotype (Fig. 5) and paratype in USNM (Type No. 44326).

#### Type locality.

Cuba: Prov. Sancti Spíritus: Buenos Aires, Trinidad Mts. 2350−2800 ft.

#### Specimens examined.

Cuba: Prov. Sancti Spíritus: Buenos Aires, Trinidad Mts., May 4 1932, SC Bruner, A Ohro, EEA. de Cuba No. 9873, 2350−2800 ft. (USNM: holotype, paratype, type No. 44326); 25 June 1940, Folk, ex F Monrós collection (USNM: 1). Buenos Aires, Trinidad Mts., May 8–14 1936, 2500–3500 ft. Darlington (MCZ: 1); Topes de Collantes, Sierra de Trinidad, June 11 1959, MW Sanderson C59–25 (INHS: 1).

#### Diagnosis.

*Stoiba fascicollis* (Figs 20–22) is similar to *Stoiba marginata* but it differs by the black pronotal coloration surrounded by a brown margin, unicolored elytra, pronotal base as broad as elytral base, fully developed hind wing and proximal 1/3 of pro-femur and proximal half of meso- and meta-femur black.

#### Description. 

Adult (n=4) length 8.3–8.7 mm, width 7.5–7.6 mm. Body (Figs 20–22) oval; elytral base as broad as pronotal base in dorsal view; profile moderately convex, highest at anterior 1/3 of elytra. Antenna (Figs 22, 50) brown with antennomeres I and II reddish and polished. Mandible (Fig. 58) with 5 teeth, maxillary palpus (Fig. 60) and labial palpus (Fig. 62) compact. Pronotum (Fig. 20) hemispherical with anterior margin slightly emarginate; dorsal surface slightly convex, more coarsely punctate than elytra with black coloration in middle; margin area brown, shallowly and sparsely punctate. Scutellum shiny black. Elytra (Fig. 21) moderately convex, black with blue luster, finely and coarsely punctate; explanate margin broadest at middle, narrower posteriorly, not extending to terminal end. Hind wing fully developed. Pro-femur brown with proximal 1/3 black; meso- and meta-femur brown with proximal 1/2 black. Spermatheca (Fig. 74) falcate with 2 openings; receptacle 1/3 as long as pump; spermathecal duct long and coarsely coiled.

#### Distribution.

Cuba: Prov. Sancti Spíritus.

### Stoiba fimbrialis

(Suffrian, 1868)

http://species-id.net/wiki/Stoiba_fimbrialis

Chelymorpha fimbrialis Suffrian 1868: 241 [original description]; Gemminger and Harold 1876: 3640 [catalog]; Leng and Mutchler 1914: 458 [list of the West Indies Coleoptera]; Spaeth 1914: 58 [catalog]; Blackwelder 1946: 745 [checklist]; Wilcox 1975: 152 [checklist].
Stoiba fimbrialis : Borowiec 1999: 130 [catalog]; Takizawa 2003: 106 [checklist]; Borowiec and Świętojańska 2012 [online catalog].

#### Type material.

Unknown

#### Type locality.

“Cuba”

#### Description

**(from original description by Suffrian 1868).** Adult length 5.7 mm, width 3.8 mm. Body oval, convex, blue. Head, antennae, pronotum, legs, elytral lateral margin dusky, densely and deeply punctured. The background color is deep blue; venter is black; head (except for black eyes), antennae, lateral margin of pronotum (moderately broad, curved upward) and legs (except for infuscate base) yellowish red; posterior edges of ventrites yellowish red and tan with posterior edge slightly raised. Antennae shorter and stockier than other Stoiba species, pronotum shiny, sparsely punctate with medio-longitudinal depression. Scutellum short and broad, bluish black with brown center. Elytra densely and coarsely punctate, sieve-like (as surface of a thimble) with lateral margin extended beyond discal edge and having discontiguous wrinkles. Claws tanned, hook-shaped with strong basal teeth.

#### Distribution.

**“**Cuba”

#### Remark.

According to the original description, the coloration and surface of *Stoiba fimbrialis* is very similar to *Stoiba marginata*. However, the type specimen is missing and no figures of *Stoiba fimbrialis* exist. It is possible that they are the same species. In this case, *Stoiba marginata* would be a junior synonym of *Stoiba fimbrialis*.

### Stoiba flavicollis

(Klug, 1829)

http://species-id.net/wiki/Stoiba_flavicollis

Figures 23–25

Cassida flavicollis Klug 1829: 14 [original description as key couplet 204].
Chelymorpha flavicolli s: Boheman 1854: 25 [description], 1856: 75 [checklist], 1862: 199 [checklist]; Suffrian 1868: 238 [description]; Gemminger and Harold 1876: 3640 [catalog]; Leng and Mutchler 1914: 458 [list of the West Indies Coleoptera].
Stoiba flavicollis : Spaeth 1909: 720 [catalog], 1914: 51 [catalog]; Blackwelder 1946: 743 [checklist]; Wilcox 1975: 151 [checklist]**;**Medvedev 1993: 9 [checklist]; Borowiec 1996: 229 [faunistic record], 1999: 130 [catalog], 2002: 116 [checklist]; Takizawa 2003: 106 [checklist]; Borowiec and Świętojańska 2012 [online catalog].
Chelymorpha nigripennis : Sturm 1826: 151 [nomen nudum].

#### Type material.

Syntypes (6) (one pictured in Figs 23–25), each with label “14169” [Klug’s collection], each with red label added “SYNTYPE, *Cassida flavicollis* Klug 1829, det. by C. Shin 2012”, deposited in ZMHB.

#### Type locality.

“Cuba”

#### Specimens examined

**(with fully developed wings).** Cuba: 14169 [Klug’s collection] (ZMHB: six syntypes); Isla de la Juventud: June 29 1921 (AMNH: 1); Prov. Ciego de Ávila: Jaronu Camaguey, Oct. 20 1934, LC Scamuzza, Bushes (USNM: 1); La Havana: ex K Kaab collection, 1916 (USNM: 3); ex FC Bowditch collection (MCZ: 5); Prov. Pinar del Río: Las Anima, S. 1500 ft. Sept. 3−5 1934, SC Bruner and AR Otero (USNM: 3); Rangel, 1935, Zayas-Garcia (UWCP: 4); Vinales, Sept. 16−22 1913 (AMNH: 1); Vinales, May 14 1913 (USNM: 1); Vinales, San Vincente, July (USNM: 1); Sierra de los Organos Vinales, 16 Jan. 1967 (UWCP: 1); Soroa, July 6−7 1974, Z and M Meszaros (UWCP: 1); Sierra Anafe, Nov. 20 1932 (MMUE: 1, USNM: 1); Sierra Anafe, July 23 1932 (MCZ: 2); Sierra Rangel, 500−1000 ft. Aug. 28−30 1927 (USNM: 1); Aspiro-Rangel, June 16 1959, NW Sanderson, C59−28 (INHS: 3); San Blas WM Mann, 1918 (USNM: 2); Sierra Rangel, 1500 ft. Aug. 29 1927 (USNM: 1); Santiago de Cuba: Gran Piedra, June 29 1955, Otero, AFA. (AMNH: 1); Loma del Gato, Sierra del Cobre, 2600−3325 ft. Sept. 24−30 1935, J Acuña, SC Bruner, LC Scaramuzza, EEA. Cuba Ento Na.10643 (USNM: 1); Bito de Cardero Turquino, June 1964, Zayas-Garcia (UWCP: 1); Loma de Gato Sierra Maestra, May 26−28 1959 (INHS: 1); Loma de Gato Sierra del Cobre, Sept. 24−30 1935 (USNM: 1); Pico Turquino, June 1936, Darlington (USNM: 3); Villa Clara: Piedra Gorda WM (USNM: 1); Gortham acc. 68498 (USNM: 1); ex Geittner collection (HNHM: 1); ex Em Friv collection (HNHM:1): no further data (BMNH: 4; MCZ: 1; FMNH: 1; MMUE: 5; MZH: 10; NHRS: 5; USNM: 1; ZNHB: 17); Mexico: Yucatan: GF Gaumer (SEMC: 3).

#### Specimens examined 

**(with brachypterous wings).** Cuba: Prov. Sancti Spíritus: Jarahueca Ote. July 14–18 1927, SC Bruner (USNM: 1); Santiago de Cuba: Gran Piedra 1100 m (HNHM: 1); Loma de Gato, Range, July 3–7 1936, 3000 ft. (MCZ: 4); Loma del Gato, Sierra del Cobre, 2600–3325 ft. Sept. 24–30 1935, ex F Monrós collection (USNM: 7); Pico Turquino, South side, 1000 ft. June 1936 (USNM: 1); Pico Turquino, South side, 30 May 1985 (UWCP: 1); Sierra Maestra, July 10–20 1922, 600–900 m, CH Ballou and SC Bruner (USNM: 1); Loma de Cala to Pico Palma Mocha, Sierra Maestra, 3600–3900 ft. May 16 1948, J Acuna (USNM: 1); Loma Cardero Pico Turquino, Aug. 1 1935, J Acuna Col. (USNM: 1); Pinares 1918 WM Mann (USNM: 1); no further data (MCZ: 2, MMUE: 2, ZMHB: 2); Mexico: Yucatan: GF Gaumer (SEMC: 1).

#### Diagnosis.

*Stoiba flavicollis* is a widely distributed species with many morphological variations such as pronotal shape (trapezoidal, rarely hemispherical without angle at postero-lateral 1/3), elytra explanate margin (broadest between anterior 1/3 and middle), elytral color (brownish black to black, often with metallic luster), and hind wing (fully-developed or brachypterous). It is mainly distinguished from *Stoiba bruneri* by pale antennomere XI, from *Stoiba clarildae* by elytra shape and coloration with metallic luster, and from *Stoiba indivisa* by distinct posterior half of elytra margin and black elytra with weak metallic luster.

#### Description.

Adult (n=118) length 6.8−9.4 mm, width 5.6−7.5 mm. Body oval (Figs 23–25), slightly or distinctly discontinuous between pronotum and elytra in dorsal view (individual variation); profile moderately convex, highest between anterior 1/3 of elytra and middle. Antennae (Figs 23, 51) reaching elytral base, brown to pale brown; antennomeres I, III and XI same in length, 2.5 times long than II; V−XI pubescent with long setae on each antennomere apex. Mandible (Fig. 58) with 5 teeth. Maxilla (Fig. 60) compact; palpifer weakly sclerotized; palpomere I 0.5 times as long as palpifer with setae apically; II 1.5 times as long as palpifer with apical region setose; III 0.8 times as long as II, setose apically; palpomere IV 1.2 times as long as III, setose with sensilla structure on apex. Labium (Fig. 62) compact; prementum subquadrate with anterior edge notched; ligula half oval with long setae; labial palpus 3-segmented; palpomere I triangular with long setae; II 2 times as long as I with long setae sub-apically; III more sclerotized than I and II with short setae and sensilla structure on apex. Pronotum (Fig. 23) hemispherical with anterior margin linear or slightly emarginate; lateral margin rounded or slightly angled or slight expanded without angle; disc moderately distinct, slightly convex; surface scale-like, smooth; lateral margin shallowly and sparsely punctate, or rarely smooth. Procoxal process (Figs 25, 64) black with hypomeron brown. Scutellum blackish brown to black. Elytra (Fig. 24) moderately convex, often bluish black to black, rarely brownish black, finely punctate; margin broadest between anterior 1/3 and middle, narrower posteriorly. Hind wing fully developed (Fig. 92) or brachypterous (Fig. 93). Legs (Fig. 25) brown except for coxae, trochanters, base of pro- and meso-femur and proximal 1/4 of meta-femur dark brown to black. Male genitalia (Figs 68–69) curved, laid laterally (deversement); aedeagal base piece rounded with membranous structure, terminal end pointed; tegmen Y-shaped; spicule V-shaped with jointed part slightly extended; ejaculatory duct loosened. Spermatheca (Fig. 75) falcate; receptacle round with 2 openings; pump area 4 times as long as receptacle.

#### Distribution.

Cuba: Isla de la Juventud; Prov. Ciego de Ávila;Pinar del Río; Santiago de Cuba; Villa Clara; Mexico: Yucatan.

#### Remarks.

*Stoiba flavicollis* is well distributed over a large area of Cuba. It is the only species with both fully developed and brachypterous wings (Figs 92–93) in our study. In the present study, we located four adult specimens collected by GF Gaumer (Dr. George Franklin Gaumer, American botanist, 1850–1929) in Yucatan, Mexico, deposited in the SEMC, and identified as *Stoiba flavicollis*. We confirmed this species identification; three of Gaumer’s four specimens have fully developed wings and one is brachypterous. This finding represents the possible extension of a range for *Stoiba* from the Caribbean islands to the Mexican mainland. However, there is no further known record of *Stoiba flavicollis* from Yucatan or other Mexican regions.

### Stoiba fuscicornis

Blake, 1966

http://species-id.net/wiki/Stoiba_fuscicornis

Figs 6 26–28

Stoiba fuscicornis Blake 1966: 219 [original description including figure]; Wilcox 1975: 151 [checklist]; Borowiec 1999: 131 [catalog]; Takizawa 2003: 106 [checklist]; Borowiec and Świętojańska 2012 [online catalog].

#### Type material.

Holotype (Fig. 6) and four paratypes in USNM; five paratypes in IJSM.

#### Type locality. 

“Jamaica”

#### Specimens examined.

Jamaica: Aug 9 1941, LV Burns (USNM: holotype, four paratypes, type No. 68196); St. Thomas Corn Puss Cap, Aug. 1941, CB Lewis (BMNH: 3).

#### Diagnosis.

*Stoiba fuscicornis* (Figs 26–28) is one of the two Jamaican species (with *Stoiba swartzii*). It is easily distinguished from *Stoiba swartzii* by leathery brown or reddish brown coloration of pronotum and elytra (not opalescent), and distinguished from *Stoiba angusticollis* by rounded body shape, finely and coarsely punctate surface, mandible with 4 teeth (Fig. 59), and elongate labial palpus (Fig. 61).

#### Description.

Adult (n=8) length 6.4−8.0 mm, width 5.6−6.7 mm. Body (Figs 26–28) round; profile moderately convex, highest between anterior 1/3 of elytra and middle. Body color leathery brown to reddish brown (not opalescent); antennae (Figs 28, 52) pale brown to dark brown. Antenna (Fig. 52) reaching elytra, pale brown to dark brown; antennomere II shortest, 0.5 times as long as III or slightly shorter; III 2 times as long as broad, as long as IV or slightly longer; III−VII gradually broader; VII as long as broad; V−XI pubescent. Mandible (Fig. 59) with 4 teeth (rarely with vestigial tooth ventrally). Maxilla (Fig. 61) elongated; palpomere IV setose with fine sensilla structure on apex. Pronotum (Fig. 26) hemispherical with anterior margin slightly emarginate, slightly angled antero-laterally; disc moderately distinct, slightly convex, shiny, smooth or finely punctate; margin area moderately punctate. Scutellum shiny brown, same as pronotum. Elytra (Figs 26–27) finely punctate; explanate margin narrower posteriorly, vague in posterior 1/3.

#### Distribution.

Jamaica: St. Thomas.

### Stoiba indivisa

Blake, 1930

http://species-id.net/wiki/Stoiba_indivisa

Figures 7 29–31

Stoiba indivisa Blake 1930: 218 [original description including figure]; Blackwelder 1946: 743 [checklist]; Wilcox 1975: 151 [checklist]; Borowiec 1996: 229 [faunistic record], 1999: 131 [catalog]; Takizawa 2003: 106 [checklist]; Borowiec and Świętojańska 2012 [online catalog].

#### Type material.

Holotype (Fig. 7) and paratype in USNM (Type No. 43116); paratype (AMNH with USNM label, Type No. 43116).

#### Type locality.

Cuba: Prov. Guántanamo.

#### Specimens examined.

Cuba: Prov. Guántanamo: WM Mann, 1918 (USNM: holotype, type No. 43116); June 22 1910, at light (AMNH: paratype, type No. 43116); ex H Rolle collection (MMUE: 1); ex Donckier collection (MMUE: 1); Prov. Santiago de Cuba: Alto de Cardero, Turquino, VI 1964, Zayas-Garcia (UWCP: 1).

#### Diagnosis.

*Stoiba indivisa* (Figs 29–31) is distinguished from *Stoiba bruneri* by pale antennae, from *Stoiba clarildae* by moderately convexprofile, and from *Stoiba flavicollis* by hemispherical pronotum without anterior margin angle. It also differs by a more emarginate pronotum anterior margin than other species and by elytra dark blue (in naked eyes) to purple (under microscope) with posterior half of margin indistinct.

#### Description.

Adult (n=5) length 6.5−6.7 mm, width 5.5−5.7 mm. Body (Figs 29–31) oval, widest at near middle in dorsal view. Head, antennae, pronotum, and legs brown with pro- and meso-femur proximal end black; meta-femur brown with proximal 1/3 black; elytra dark blue to purple and slightly opalescent. Head withdrawn into prothorax except entire eyes and epicranial suture exposed in dorsal view; inter-ocular area 2 times as broad as eye, slightly depressed medially; maxillary palpus (Fig. 60) and labial palpus (Fig. 62) compact. Pronotum (Figs 7, 29) hemispherical, brown with base line black; base 2 times as broad as anterior edge; anterior edge moderately emarginate, anterior 2/3 of lateral edge gradually broader, curved at posterior 1/3; margin moderately defined, sparsely punctate. Procoxal process (Fig. 31) slightly convex, shiny, black. Scutellum triangular, shiny black. Elytra (Fig. 30) moderately convex with surface scale-like, shiny, finely and coarsely punctate; umbone moderately projected and angled; margin distinct in anterior half and vague in posterior half. Hind wing fully developed. Legs (Fig. 31) brown with coxae, trochanters, proximal end of pro- and mesofemur, proximal 1/3 metafemur black.

#### Distribution.

Cuba: Guántanamo; Santiago de Cuba.

### Stoiba lurida

(Suffrian, 1868)

http://species-id.net/wiki/Stoiba_lurida

Chelymorpha lurida Suffrian 1868: 240 [original description]; Gemminger and Harold 1876: 3640 [catalog]; Leng and Mutchler 1914: 458 [list of the West Indies Coleoptera]; Spaeth 1914: 59 [catalog]; Blackwelder 1946: 745 [checklist]; Wilcox 1975: 152 [checklist].
Stoiba lurida : Borowiec 1999: 131 [catalog]; Takizawa 2003: 106 [checklist-misspelled]; Borowiec and Świętojańska 2012 [online catalog].

#### Type material. 

Unknown

#### Type locality.

“Cuba”

#### Description

**(from original description by Suffrian 1868).** Adult length 9.0 mm, width 7.4 mm. *Stoiba lurida* is generally similar to large size of *Stoiba flavicollis*, but *Stoiba lurida* shows brownish yellow pronotum when it is dried and shows gold shimmering in fresh specimens; pronotal and elytral margins more coarsely and roughly punctate. Sometimes it was identified as *Calligrapha spiraeae* (Say) in North America. Body slightly oval, convex, broadest within anterior half of elytra, dark reddish brown, opalescent with punctures on lateral margins of pronotum and elytra. Pronotum with posterior margin broad, flat; disc finely and sparsely punctate, lateral margin slightly more punctate. Elytra with posterior half of lateral margin not clearly explanate; lateral decline margin area slightly more punctate than raised edge area. Ventral surface (abdomen) brownish yellow to reddish yellow.

#### Distribution.

**“**Cuba”

### Stoiba marginata

Blake, 1934

http://species-id.net/wiki/Stoiba_marginata

Figures 8 32–34

Stoiba marginata Blake 1934: 53 [original description including figure]; Blackwelder 1946: 743 [checklist]; Wilcox 1975: 151 [checklist]; Borowiec 1999: 131 [catalog]; Takizawa 2003: 106 [checklist]; Borowiec and Świętojańska 2012 [online catalog].

#### Type material.

Holotype and paratype in USNM (Type No. 44325).

#### Type locality.

Cuba: Prov. Sancti Spíritus, Buenos Aires, Trinidad Mts., 2350–2800 ft.

#### Specimens examined.

Cuba: Prov. Sancti Spíritus, Buenos Aires, Trinidad Mts., 2350–2800 ft. May 4 1932, SC Bruner, Otero. EEA de Cuba No. 9872. (USNM: holotype, paratype, type No. 44325); June 17–23 1939, CT Parsons (MCZ: 1).

#### Diagnosis.

*Stoiba marginata* (Figs 32–34) differs from *Stoiba fascicollis* by the elytral base broader than the pronotal base, black pronotal coloration extending to the base, prosternum black, coarsely punctate dorsal surface of the pronotum and elytra, black elytral disc with coloration extending to antero-lateral region of marginal area and femora black over their proximal half.

#### Description.

Adult (n=3) length 8.2−8.4 mm, width 7.6−7.8 mm. Body (Figs 32–34) rounded, widest and highest near middle. Color of head, antennae, pronotum margin area and elytra margin area brown except for black antero-lateral region of elytra margin; legs brown with coxae, trochanters and over proximal half of femora black. Head withdrawn into prothorax, up to half of eyes in dorsal view; inter-ocular area 2 times as broad as eye, flat with cranial suture and antennal sockets medially; maxillary palpus (Fig. 60) and labial palpus (Fig. 62) compact. Pronotum hemispherical with anterior edge slightly emarginate; surface coarsely and roughly punctate; base 2.2 times as broad as anterior edge; disc well defined by black coloration surrounded brown margin area except for anterior region. Prosternum (Fig. 34) black with brown hypomeron. Scutellum triangular, black, scale-like or occasionally punctate. Elytra (Fig. 33) moderately convex with surface coarsely and roughly punctate; discal area defined by black coloration with black coloration extending to middle of margin area; umbone slightly projected; margin area mainly brown, narrower posteriorly, extending to rear end. Hind wing brachypterous. Legs (Fig. 34) mainly brown with coxae, trochanters, and over proximal half of femora black. Spermatheca (Fig. 77) falcate with two openings, gradually narrower; receptacle area narrow and not well defined; spermathecal duct loosely coiled.

#### Distribution.

Cuba: Sancti Spíritus.

### Stoiba nigricans

Zayas, 1939

http://species-id.net/wiki/Stoiba_nigricans

Figures 9 35–37

Stoiba nigricans Zayas 1939: 255 [original description including figure]; Blackwelder 1946: 743 [checklist]; Wilcox 1975: 151 [checklist]; Borowiec 1999: 131 [catalog]; Takizawa 2003: 106 [checklist]; Borowiec and Świętojańska 2012 [online catalog].

#### Type material.

Holotype in USNM (Type No. 53530).

#### Type locality.

Cuba: Prov. Santiago de Cuba: Loma Gato, Clemente.

#### Specimens examined.

Cuba: Prov. Santiago de Cuba: Loma Gato, Clemente, July 1938 (USNM: holotype No. 53530); Sierra Maestra 800–4000 m, Aug 7 1929, Frere Clement, ex FC Monrós collection (USNM: 1).

#### Diagnosis.

*Stoiba nigricans* (Figs 35–37) is distinguished by dark antennae, brownish black pronotum and elongate, C-shaped spermatheca, these serving to separate it from *Stoiba bruneri* and *Stoiba clarildae*. It also differs from *Stoiba bruneri* by the unicolored antennae and trapezoidal pronotum, and from *Stoiba clarildae* by the pronotal base as broad as the elytral base.

#### Description.

Adult (n=2) length 8.8–9.0mm, width 6.9–7.0mm. Body (Figs 35–37) oval, broadest between anterior 1/3 and middle of elytra in dorsal view; profile moderately convex, highest at middle of elytra. Antennae (Figs 35, 55) brownish black with antennomeres VI–XI pubescent with pale setae; antennomeres I–IV shiny, glabrous; antennomere III 3 times as long as II, 1.3 times as long as IV. Mandible (Fig. 58) with 5 teeth, maxillary palpus (Fig. 60) and labial palpus (Fig. 62) compact. Pronotum (Fig. 35) brownish black, trapezoidal with anterior edge linear; antero-lateral edge smoothly angled, anterior 2/3 gradually broader, rounded and slightly narrower in posterior 1/3; disc slightly convex; with discal surface smooth, scale-like; lateral margin region finely and sparsely punctate. Elytra (Fig. 35) bluish black, slightly opalescent, finely punctate; margin area distinct, narrower posteriorly. Hind wing brachypterous. Spermatheca (Fig. 78) elongate, C-shaped with two openings, receptacle 0.2 times as long as pump; spermathecal duct short and coarsely coiled.

#### Distribution.

Cuba: Prov. Santiago de Cuba.

### Stoiba oteroi

Zayas, 1952

http://species-id.net/wiki/Stoiba_oteroi

Stoiba oteroi Zayas 1952: 72 [original description includes figure]; Wilcox 1975: 151 [checklist]; Borowiec 1999: 131 [catalog]; Takizawa 2003: 106 [checklist]; Borowiec and Świętojańska 2012 [online catalog].

#### Type material.

Unknown.

#### Type locality.

Cuba: Villa Clara, Lomas de Trinidad.

#### Description

**(from original description by Zayas 1952).** Adult length 8.0 mm, width 7.0 mm. Body flattened, rounded, slightly steel black with antennae, head, margin area of pronotum, and legs (except basal half of femora) dull yellow. Head with anterior half visible in dorsal view; antennae short, yellow, brighter distally; eyes black; mandible and palpi ferruginous at apex. Pronotum small, narrow, and short with discal area widely dark stained and with strongly marked longitudinal groove medially; surface sparsely and coarsely punctate, more punctate medially extending posteriorly. Scutellum shiny black. Elytra dark stained as on pronotum, symmetrically and deeply punctured with external margins slightly expanded and declined. Hind wing fully developed. Legs dull yellow with tips of tibiae and femora black.

#### Distribution.

Cuba: Prov. Villa Clara.

### Stoiba swartzii

(Thunberg in Schönherr 1808)

http://species-id.net/wiki/Stoiba_swartzii

Figures 38–43

Cassida swartzii Thunberg in Schönherr 1808: 229 [original description with figure]; Sekerka 2008: 305 [Thunberg’s collection list in Uppsala University Zoological Museum].
Chelymorpha swartzii : Boheman 1854: 26 [description], 1856: 76 [checklist]; Leng and Mutchler 1917: 212 [supplement of previous list].
Chelymorpha swartzi : Boheman 1862: 199 [checklist]; Gemminger and Harold 1876: 3641 [catalog]; Leng and Mutchler 1914: 458 [list of the West Indies Coleoptera].
Stoiba swartzi : Spaeth 1914: 51 [catalog]; Blake 1966: 214 [figure]; Borowiec 1996: 229 [faunistic record], 1999: 131 [catalog].
Stoiba swartzii : Blackwelder 1946: 743 [checklist]; Wilcox 1975: 151 [checklist]; Chaboo 2000: 379 [outgroup in phylogenetic analysis]; Takizawa 2003: 107 [checklist]; Borowiec and Świętojańska 2012 [online catalog].
Stoiba rufa Blake 1966: 218 [original description including figure]; Wilcox 1975: 151 [checklist]; Borowiec 1999: 131 [catalog]; Takizawa 2003: 106 [checklist]; Borowiec and Świętojańska 2012 [online catalog]. syn. nov.

#### Type material.

Holotype (Figs 38–40), with label “Jamaica”, with red label added “HOLOTYPE, *Cassida swartzii* Thunberg 1808,det. by C. Shin 2012”, deposited in NHRS.

#### Type locality.

“Jamaica”

#### Specimens examined.

Jamaica: Clarendon Parish: Cumberland District, 3000 ft. Dec. 15–18 1919 (AMNH: 1);W Robinson (AMNH: 2); Kingston Parish: Dec. 1967, NLH Krauss (USNM: 1); Portland Parish: Morces Gap, July 22 1958, MW Sanderson (INHS: 1); St. Andrew Parish: Cinchona Garden in Blue Mts. on vine, June 13 1931, M Kisliuk (AMNH: 1, USNM: 21); Cinchona Garden in Blue Mts. 4900 ft. July 23 1923, FR Mason (MMUE: 1); St. Thomas Parish: Corn Puss Gap, June 1937 (CMNH: 2); Morant Bay Rd. 14.5 miles east of Kingston, 21 July 1963, TH Farr (USNM: holotype of *Stoiba rufa*, type No. 68195); Morant Bay Rd. 14.5 miles east of Kingston, Sept. 6 1964, TH Farr (USNM: paratype of *Stoiba rufa*, type No. 68195); no further data: ex Holland collection (CMNH: 2); F Klages, ex Holland collection (CMNH: 1); “5433C” (BMNH: 1); “45 100” [1845 Jamaica, purchased from Gosse ] (BMNH: 1); “1867, 6756” [67–56, acquired in 1867] (BMNH: 2); ex Baly collection [retained by Spaeth] (MMUE: 1); no further data (NHRS: holotype; UWCP: 2).

#### Diagnosis.

*Stoiba swartzii* (Figs 10, 38–43) is distinguished from *Stoiba angusticollis* and *Stoiba fuscicornis* by its brown pronotum and reddish and opalescent elytra. It also differs from *Stoiba angusticollis* by its 4-toothed mandible (Fig. 59), elongate maxillary palpus (Fig. 61), and elongate labial palpus (Fig. 63) and from *Stoiba fuscicornis* by a moderately distinct elytral margin.

#### Description.

Adult (n=42) length 5.2–10.3 mm, width 5.0–9.6 mm. Body (Figs 10, 38–43) rounded to oval, broadest at anterior 1/3 of elytra in dorsal view; profile moderately convex, highest at anterior 1/3 of elytra. Antennae (Figs 38, 56) pale brown; antennomere III 2 times as long as II; IV as long as III or slightly shorter; V–XI pubescent; VII as long as broad; XI as long as antennomere I. Mandible (Fig. 59) with four distinct teeth with one vestigial tooth ventrally or absent; maxillary palpus (Fig. 61) and labial palpus (Fig. 63) elongated. Pronotum (Figs 38–43) hemispherical or trapezoidal with anterior margin slightly emarginated or linear. Scutellum shiny, yellowish brown to deep red (coloration same as pronotum or elytra). Elytra reddish brown to deep red, finely punctate, distinctly opalescent (blue); elytra margin area often entirely distinct. Hind wing fully developed.

**Figures 44–63.**
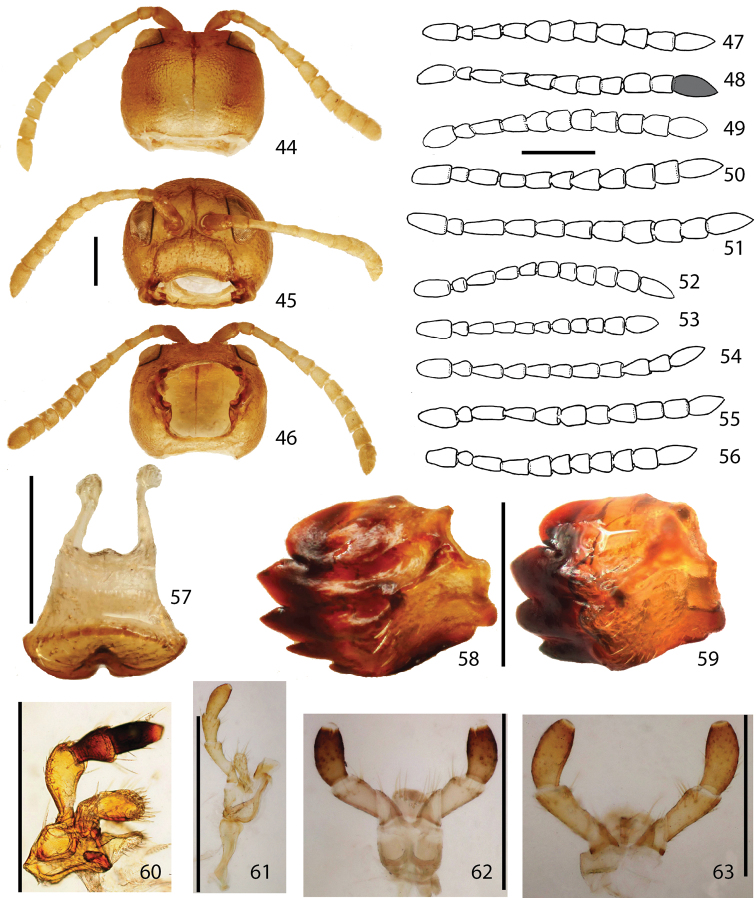
Head of *Stoiba flavicollis*. **44** dorsal view **45** anterior view **46** ventral view **47–56** antennae **47** *Stoiba angusticollis* **48** *Stoiba bruneri* **49** *Stoiba clarildae* **50** *Stoiba fascicornis* **51** *Stoiba flavicollis* **52 ***Stoiba fuscicornis* **53** *Stoiba indivisa* **54** *Stoiba marginata* **55** *Stoiba nigricans* **56** *Stoiba swartzii* **57–63** mouth parts **57** labrum (*Stoiba flavicollis*) **58** mandible (*Stoiba flavicollis*) **59** mandible (*Stoiba swartzii*) **60** maxilla (*Stoiba flavicollis*) **61 **maxilla (*Stoiba swartzii*) **62** labium (*Stoiba flavicollis*) **63** labium (*Stoiba swartzii*). (scale bar = 1.0 mm)

**Figures 64–80.**
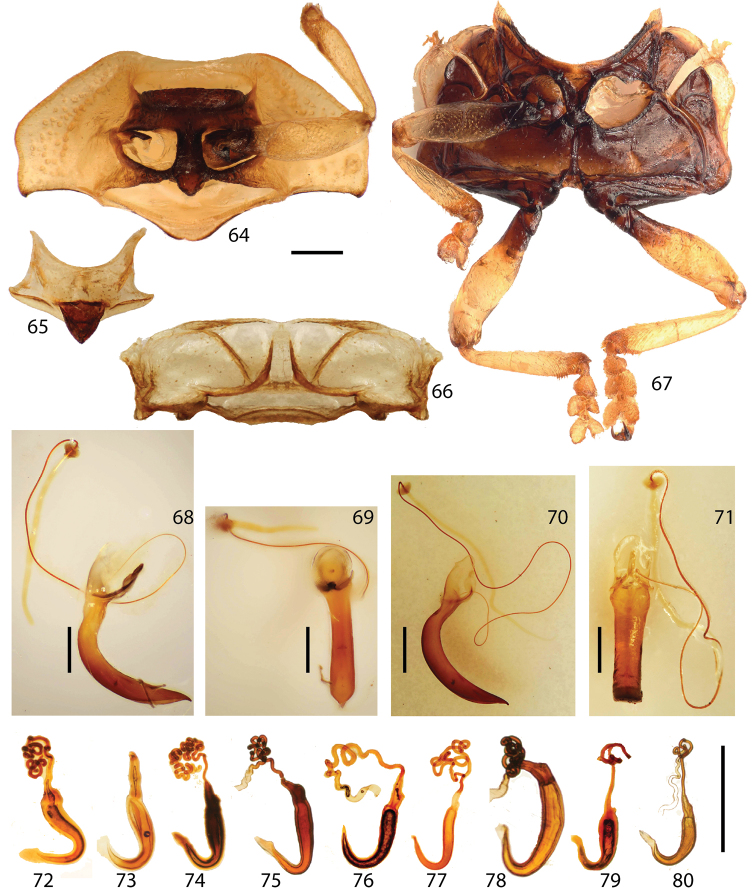
Thorax. **64** prosternum **65** mesonotum **66** metanotum **67**, meso-, metathoracic sterna **68–69** male genitalia (*Stoiba flavicollis*) **70–71** male genitalia (*Stoiba swartzii*) **72–80** Spermathecae **72** *Stoiba angusticollis* **73** *Stoiba bruneri* **74** *Stoiba fascicornis* **75** *Stoiba flavicollis* **76** *Stoiba fuscicornis* **77** *Stoiba marginata* **78** *Stoiba nigricans* **79** *Stoiba swartzii* **80** *Stoiba rufa* (=*Stoiba swartzii*). (scale bar = 1.0 mm)

**Figures 81–91.**
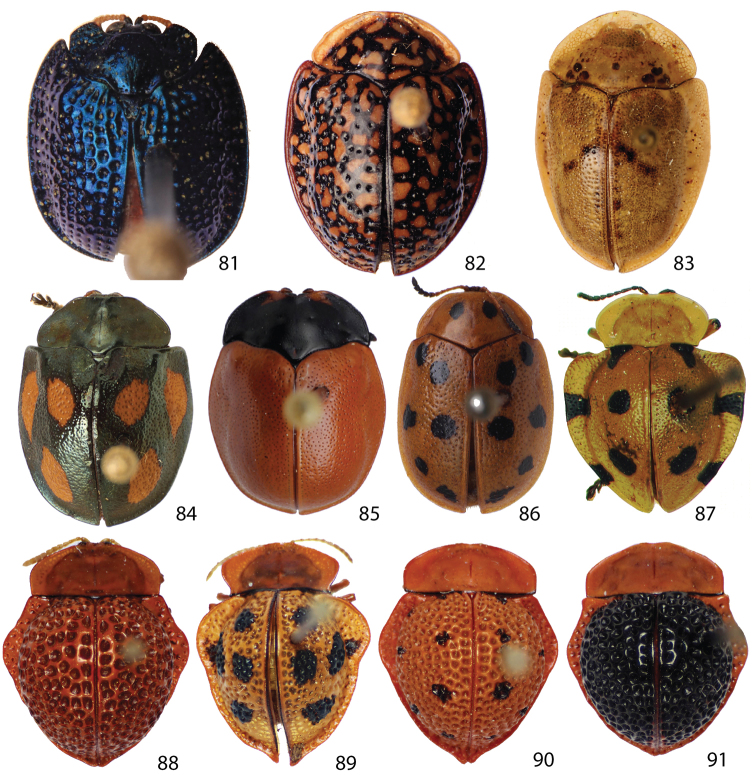
Outgroup **81** *Spaethiella* sp. **82** *Asteriza flavicornis* **83** *Physonota alutacea* **84** *Stolas* sp. **85** *Chelymorpha* sp. **86** *Phytodectoidea* sp. **87** *Elytrogona bulla* **88** *Elytrogona Bacca* **89** *Elytrogona gemmata* **90** *Elytrogona nigrodorsata* **91** *Elytrogona quatuordecimmaculata*.

#### Distribution.

Jamaica: Clarendon Parish; Kingston Parish; Portland Parish; St. Andrew Parish; St. Thomas Parish.

#### Nomenclature.

Borowiec (1996) indicated Boheman (1862) as the author of *Stoiba swartzii*. Sekerka (2008) clarified Thunberg as the author and the year (1808). In the original description (Thunberg in Schönherr 1808), Thunberg described with both named *Cassida swartzi* and *Chelymorpha swartzii*. According to ICZN [Article 32.2.1], “If a name is spelled in more than one way in the work in which it was established, then, except as provided otherwise in this Article, the correct original spelling is that chosen by the First Reviser [Art. 24.2.3] (or, if applicable, by an original author when acting as First Reviser [Art. 24.2.4]).” Therefore, the correct name is *Stoiba swartzii* which was used by the first reviser, Boheman (1854).

#### Remarks.

The type specimen of *Stoiba swartzii* is included in the catalog of Thunberg’s collection at UUZM, however the physical location of the specimen is within Schöenherr‘s collection at NHRS. Thunberg described *Stoiba swartzii* mainly by coloration. He mentioned the scutellum with the same color as the elytra. We found the color of scutellum can be the same color of either the pronotum or the elytra. Blake (1966) distinguished *Stoiba swartzii* from *Stoiba rufa* by mentioning the coloration of *Stoiba swartzii* as having “deep purple blue or even deep reddish blue elytra.” It needs to be clarified that the purple or blue coloration is not the basic color of elytra but is from opalescence. Blake (1966) also used body size, proportion, and other coloration to distinguish between *Stoiba swartzii* and *Stoiba rufa*. During our study, we found that different sized specimens of *Stoiba swartzii* have different color variations. We also found varia- tion in body proportion, elytral width, and pronotum shape (Figs 10, 38, 41–43). The figure of *Stoiba swartzii* in the original description (Thunberg in Schönherr 1808) shows a rounded body shape, which is different from the oval body shape on the holotype of *Stoiba rufa* (Fig. 10). However, the paratype of *Stoiba rufa* (Fig. 41), which is from the same collecting event and designated by Blake (1966), looks more similar to other *Stoiba swartzii* (Figs 38, 42–43). After comparing the holotypes of *Stoiba swartzii* and *Stoiba rufa* (Fig. 10), and other specimens,including a female genitalia comparison (Figs 79–80), we concluded that the variation between *Stoiba swartzii* and *Stoiba rufa* is continuous. Therefore, we synonymized *Stoiba rufa* with *Stoiba swartzii*.

## Discussion

**Figures 92–105.**
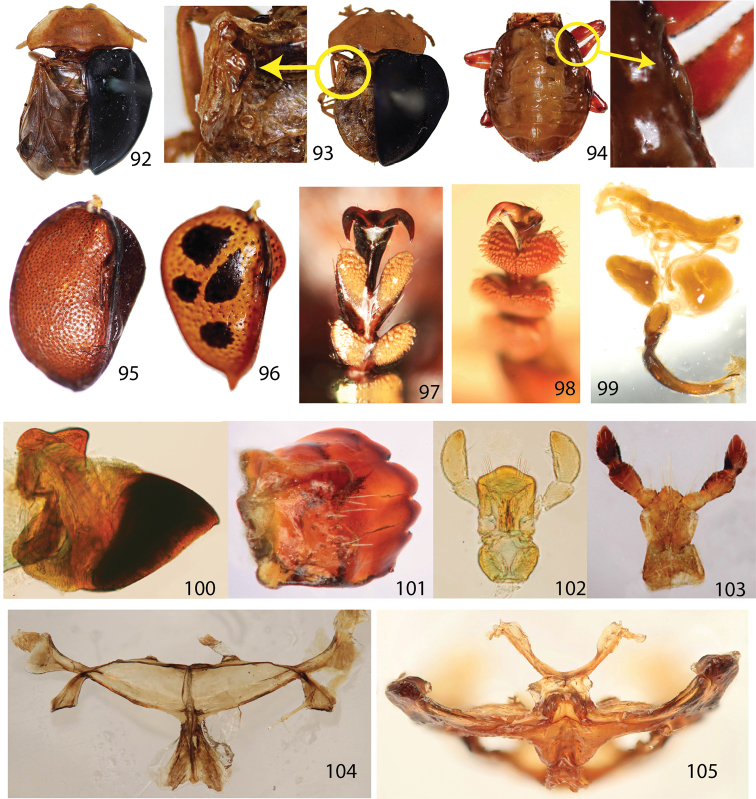
Hind wings **92** fully developed hind wing (*Stoiba flavicollis*) **93** brachypterous wing (*Stoiba flavicollis*) **94** vestigial wing (*Elytrogona gemmata*) **95–96** ventral surface of elytra **95** *Stoiba flavicollis* **96** *Elytrogona gemmata*. **97–98** basal tooth of claw **97** *Stoiba flavicollis* **98** *Elytrogona gemmata* **99** male genitalia of *Asteriza flavicornis* **100** mandible (*Spaethiella* sp.) **101** mandible (*Asteriza flavicornis*) **102** labium (*Spaethiella* sp.) **103** labium (*Asteriza flavicornis*) **104** metendosternite (*Stoiba flavicollis*) **105** metendosternite (*Elytrogona gemmata*).

**Figure 106.**
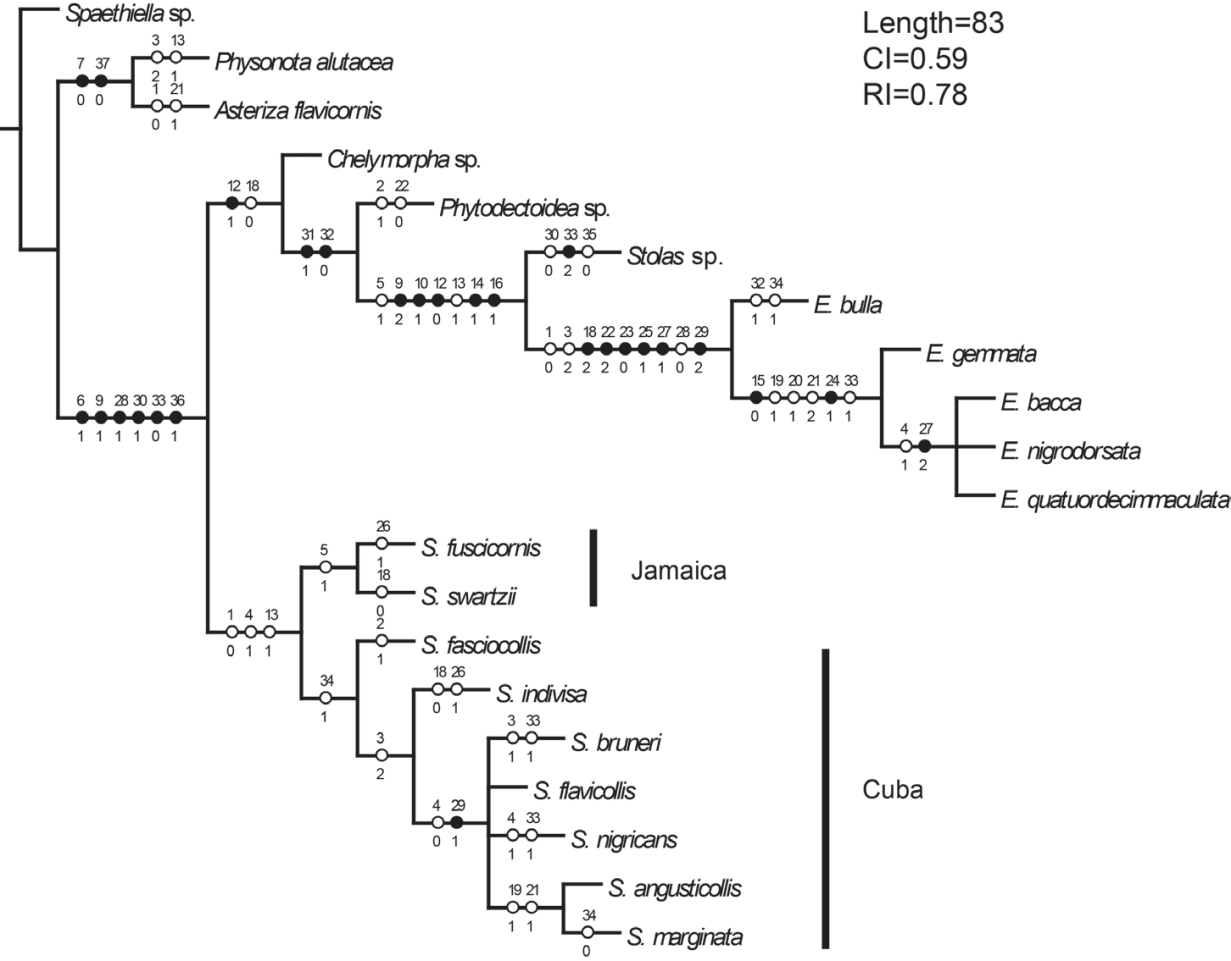
Consensus tree.

**Phylogeny.** Parsimony analysis found the four most parsimonious trees with 83 steps (CI=0.59, RI=0.78). Monophyly (Fig. 106) of the genus *Stoiba* is supported by three characters—pale yellow antennae (#1), antennomere VII broader than its length (#4), and rounded basal line of pronotum (#12). The pale coloration of antennae (#1) was hypothesized as a possible synapomorphy of genera *Stoiba* and *Elytrogona* based on the identification keys by Borowiec and Świętojańska (2012). Our study shows that pale antennae evolved independently in *Stoiba* and *Elytrogona*. *Stoiba* and *Elytrogona* were thought to be sister taxa, or *Elytrogona* was thought to be a derived group from *Stoiba* because of vestigial hind wing (Blake 1930; Chaboo 2007). For the phylogenetic analysis, we made three character states [fully developed (Fig. 92), brachypterous (Fig. 93), and vestigial (Fig. 94)] for the hind wing modification. According to our analysis, *Stoiba* and *Elytrogona* are not sister taxa. We show that the brachypterous wings of *Stoiba* and the vestigial wings of *Elytrogona* evolved independently in each clade. Other characters [membranous meso-, metanotum, fused thoracic notum (Fig. 94), fused elytral suture (Figs 87–91), and broad and rigid metendosternite (Fig. 105)] occurred only in *Elytrogona*. Those *Stoiba* species with brachypterous wings showed the same morphology as in other *Stoiba* species with fully developed wings.

The genus *Stoiba* is divided into two clades: seven Cuban species grouped by a tightly coiled spermathecal duct (#34) and two Jamaican species grouped by a mandible with 4 teeth or 4 teeth and vestigial teeth (#5).

Five *Stoiba* species with brachypterous wings form a clade within the seven Cuban species. Interestingly, this clade can also be supported by antennomere VII longer than its width (#4), which is a reversed character state from the monophyly of the genus *Stoiba*.

The reduced hind wing is one of the most interesting features of *Stoiba* and *Elytrogona*. Generally, it occurs because of stable habitats, metabolism efficiency, parasitism, or trade-offs (Zera 1985; Roff 1990; Wagner and Liebherr 1992; Mole and Zera 1993). We cannot tell what caused this hind wing modification. However, this hind wing modification is not related to body size or sexual dimorphism.

**Type specimens.** The depositories for types of *Stoiba angusticollis*, *Stoiba fimbrialis*, *Stoiba lurida*, *Stoiba oteroi* are unknown.

According to Horn and Kahle (1936) and Borowiec and Świętojańska (2012), the possible depositories of the type specimens of *Stoiba angusticollis*, *Stoiba fimbrialis*, and *Stoiba lurida* (Suffrian collection) are MLUH or ZMHB. We contacted both museums, but those type specimens were not located in MLUH and ZMHB.

Dr. Michael A. Ivie (Montana State University) provided photographs of the type specimen of *Stoiba barroi*, which we confirmed in several drawers of photographs of the Zayas cassidine collection taken by Marc Branham and Jennifer Zaspel (University of Florida). However, the type specimen of *Stoiba oteroi* was not found in these same drawers or in the rest of the Zayas collection of Cassidinae.

## Supplementary Material

XML Treatment for Stoiba

XML Treatment for Stoiba angusticollis

XML Treatment for Stoiba barroi

XML Treatment for Stoiba bruneri

XML Treatment for Stoiba clarildae

XML Treatment for Stoiba fascicollis

XML Treatment for Stoiba fimbrialis

XML Treatment for Stoiba flavicollis

XML Treatment for Stoiba fuscicornis

XML Treatment for Stoiba indivisa

XML Treatment for Stoiba lurida

XML Treatment for Stoiba marginata

XML Treatment for Stoiba nigricans

XML Treatment for Stoiba oteroi

XML Treatment for Stoiba swartzii
